# Hydrogen Transfer to Internal Alkynes Using Secondary Amines on Carbon-Supported Noble Metals

**DOI:** 10.1021/acsphyschemau.6c00058

**Published:** 2026-06-16

**Authors:** Katharina Konieczny, Tianyin Qiu, Jan Paul Menzel, Jacqueline Maslack, Victor S. Batista, Eszter Baráth

**Affiliations:** † 28392 Leibniz-Institut für Katalyse e.V. (LIKAT), Albert Einstein Str. 29a, Rostock D-18059, Germany; ‡ Department of Chemistry, 5755 Yale University, 225 Prospect Street, P.O. Box 208107, New Haven, Connecticut 06520, United States

**Keywords:** noble metal, carbon support, hydrogen transfer, isokinetic temperature, secondary amines

## Abstract

The catalytic hydrogen transfer of internal alkynes using carbon-supported noble metals (Pd/C, Pt/C) was systematically studied with various secondary amines, such as indoline (Ind), tetrahydroquinoline (Thq), diisopropylamine ((^i^Pr)_2_NH)), and, for comparison, diisopropylethylamine ((^i^Pr)_2_NEt)) as hydrogen donors. The reactions proceeded sequentially, forming *(Z)*- and *(E)*-olefins as interim products, having *(Z)* as a major olefin product, which were sequentially hydrogenated to alkanes. The presence of *(E)*-alkene isomers was also observed as minor products. Among the tested systems, Pt/C-Ind, Pd/C-Ind, Pt/C-Thq, and Pd/C-Thq exhibited the highest activity and selectivity. Initial reaction rates and activation parameters (activation energy, *E*_a_; enthalpy of activation, Δ*H* ^‡^; and entropy of activation, Δ*S* ^‡^) were determined for these systems. To further elucidate the reaction mechanism, density functional theory (DFT) calculations were performed. The computational results revealed that the balance between adsorption strength and binding energy of reactants and intermediates governs the observed selectivity trends. Notably, the strongly negative activation entropies suggest a rigid, highly ordered transition state, independent of the metal catalyst.

## Graphical abstract

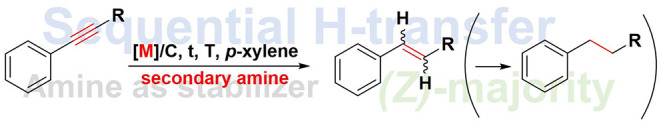

## 1 Introduction

Hydrogen transfer (HT) reactions play a critical role in the synthesis and modification of a wide range of organic compounds, including alkenes, alkynes, imines, and carbonyl derivatives. These reactions are valued for their broad applicability, often proceeding under mild conditions without the need for molecular hydrogen and offering potential for both stereoselective and enantioselective transformations.^1–3^ HT processes can be catalyzed by both homogeneous and heterogeneous systems, with the latter providing notable advantages in terms of catalyst recovery, stability, and compatibility with sequential or continuous-flow processes.^4,5^

At the core, HT reactions involve the transfer of hydrogen atoms, typically in the form of a hydride and a proton, or as two protons and two electrons, from a donor to an acceptor molecule.^1–3^ Mechanistically, two general pathways are recognized: direct hydride transfer and indirect pathways involving the formation of a metal hydride intermediate to facilitate the transfer. Compared to conventional hydrogenation using H_2_ gas, hydrogen transfer offers safer, more accessible alternatives with simpler setups and cost-effective hydrogen sources.

Secondary alcohols, such as isopropanol, are among the most widely used hydrogen donors, serving as both solvents and reductants in a dual role.^1^ Other efficient hydrogen sources include ammonium formate, sodium borohydride, and ethanolamine, which have been used successfully in the partial hydrogenation of alkynes, particularly in the presence of noble metals.^6,7^ These approaches illustrate the growing interest in leveraging alternative H-donors in catalytic hydrogen transfer reactions to enable selective and sustainable transformations.

The heterogeneously catalytic synthesis of alkenes from alkynes offers several advantages over homogeneous methods, including enhanced chemical and thermal stability, as well as simplified product separation.^1,5–11^ Selective hydrogenation of alkynes remains a key transformation in industrial chemistry. To this day, the most widely used heterogeneous catalyst for the selective, direct hydrogenation of alkynes to the corresponding *(Z)*-alkenes with molecular hydrogen is the Lindlar catalyst (5 wt % Pd/CaCO_3_ treated with Pb­(OCOCH_3_)_2_ and quinoline).^12–15^

In our previous studies, we found that tertiary amines serve as highly effective hydrogen donors in the hydrogenation of olefins and alkynes catalyzed by noble metals supported on carbon.^4,5^ For alkynes, the presence of the amine donor proved to be critical for selectivity. The donor plays a dual role, acting both as a hydrogen source and as an electronic and geometric stabilizer of surface intermediates.^4^ Theoretical analysis revealed that, despite being thermodynamically less stable, the *(Z)*-isomer can dominate as an intermediate. This is due to the formation of a surface complex between the alkyne and the amine at high amine coverage, which favors *(Z)-*selective adsorption and hydrogenation. This intermediate pathway ultimately leads to the overhydrogenated product.^4,5^ The hydrogen abstraction and donation capabilities of Pt and Pd, when combined with a suitably chosen hydrogen donor, critically shape the reaction profile and product distribution.

Building on the successful application of tertiary alkyl amines in the hydrogen-transfer reaction of alkynes,^4,5^ we expanded the amine scope to include secondary amines, using internal alkynes as model substrates. Our goal was to determine whether *N*-unsubstituted secondary amines (both cyclic and acyclic) could offer improved selectivity, more favorable activation parameters, and faster reaction rates, as previously observed for tertiary amines.

Secondary amines (cyclic or alkyl) are a common structural motif in ligand frameworks of homogeneous metal complexes,^16–19^ including noble and base metals. However, their use in heterogeneous catalytic systems has been less explored, especially in the context of direct or indirect roles in hydrogenation or hydrogen transfer.^16–19^ Notably, their hydrogen-donating ability and broad reactivity have been well documented.^20–24^

In this study, we evaluate secondary amines as reducing agents in the HT reaction of substituted alkynes, such as phenyl/carboxyl-, phenyl/phenyl-, and phenyl/methyl, on Pd/C and Pt/C catalysts. We assess reaction kinetics, turnover frequencies (TOF), and activation parameters (*E*_a_, Δ*H* ^‡^, Δ*S* ^‡^) on both metal surfaces. Using these model substrates, we investigate the molecular origin of the observed *(Z)*-selectivity and the sequential progression toward fully hydrogenated products.

DFT calculations reveal how specific binding energies and electron distributions of the starting alkynes, along with the formation of surface-bound intermediates, dictate the reaction pathway. Our computational and experimental data align closely, highlighting the role of secondary amines as effective promoters. They facilitate *(Z)*-isomer formation by acting as both hydrogen donors and electronic stabilizers throughout the reaction.

## 2 Results and Discussion

### 2.1 Experimental Details

We employed internal alkynes as model compounds (Figure 1), such as methyl phenylpropiolate (substrate **1**), diphenylacetylene (substrate **2**), and 1-phenyl-1-propyne (substrate **3**). These substrates were chosen to systematically investigate how varying substituents influence adsorption behavior as well as the electronic and steric contributions to the overall reaction profile.

**Figure 1:**
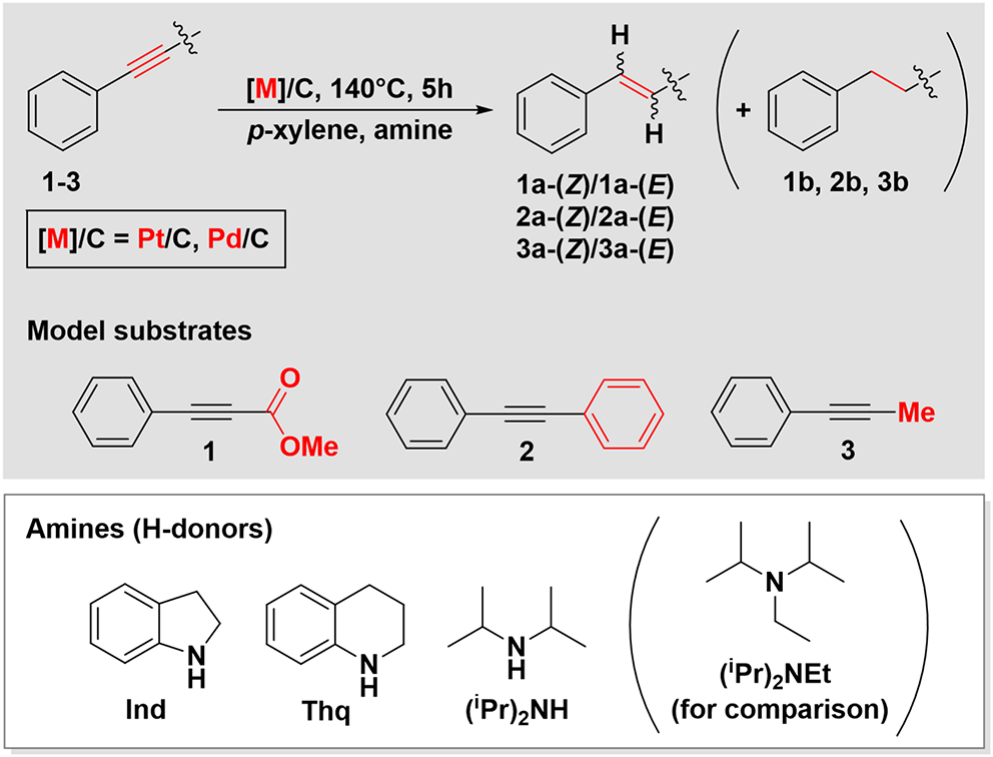
Hydrogen transfer of model alkynes **1**, **2**, **3** using secondary amines as H-donors.

Commercially available Pt/C and Pd/C catalysts with 10 wt % metal loading were used. Catalysts were freshly activated prior to the experiment, following the reduction protocol detailed in the Supporting Information (SI). The carbon-supported noble metals were characterized in terms of metal loading, metal dispersion, BET surface area, and pore volume (Table 1, SI Figures S1 and S2).

**Table 1:** Characterization of Noble Metals on Carbon

	**catalyst**		
	Pt/C	Pt/C	Pd/C
**characterization type**	(10 wt %) (A)	(10 wt %) (B)	(10 wt %)
metal loading (wt %)^a^	9.1	10.2	9.9
metal dispersion (%)^b^	22.9	30.2	16.7
metal particle diameter (nm)^b^	4.1	3.1	5.6
metal particle diameter (nm)^c^	5.0 (±2.0)	3.6 (±2.7)	5.7 (±1.1)
BET surface area/total (m^2^ g^–1^)	958	943	851
BET surface area/mesopore (m^2^ g^–1^)	336	306	168
BET surface area/micropore (m^2^ g^–1^)	622	636	683
pore volume/total (cm^3^ g^–1^)	1.35	1.21	0.77
pore volume/mesopore (cm^3^ g^–1^)	0.82	0.67	0.39
pore volume/micropore (cm^3^ g^–1^)	0.53	0.54	0.36

a Metal content was determined with ICP-OES (inductively coupled plasma optical emission spectroscopy).

b Determined by H_2_ chemisorption.

c Particle size was measured by TEM (see SI, Figure S3). (In order to cover the experimental need, we used two Pt/C batches (A and B); for the detailed description of the reaction procedures, please see the SI.)

Pt and Pd were selected as catalysts (Table 1) due to their well-established ability to abstract and donate hydrogen from liquid phase donors, as well as to mediate stepwise hydrogen transfer in substrates bearing multiple unsaturated bonds.^5,8,9^ We investigated various catalyst-donor combinations: Pt/C–Ind, Pt/C–Thq, Pt/C–(^i^Pr)_2_NH, Pt/C–(^i^Pr)_2_NEt (control), and the corresponding Pd/C-based systems (Table 2).

**Table 2:** Hydrogen Transfer of Internal Alkynes on Carbon-Supported Noble Metals in the Presence of Secondary Amines as H-Donors in *p*-Xylene as Solvent

					**yield (%)**		
entry^a^,^b^	**substrate**	**catalyst**	**amine**	**conversion (%)**	**(** ***Z*** **)-alkene (1a-(** ***Z*** **)–3a-(** ***Z*** **))**	**(** ***E*** **)-alkene (1a-(** ***E*** **)–3a-(** ***E*** **))**	**overhydrogenated product (1b–3b)**
**1**	**1**	Pt/C	Ind	100	0	0	100
**2**		Pd/C	Ind	100	0	0	100
**3**		Pt/C	Thq	100	0	6	94
**4**		Pd/C	Thq	100	0	0	100
**5**		Pt/C	(^i^Pr)_2_NH	100	3	69	27
**6**		Pd/C	(^i^Pr)_2_NH	3	3	0	0
**7**		Pt/C	(^i^Pr)_2_NEt	100	3	10	87
**8**		Pd/C	(^i^Pr)_2_NEt	7	7	0	0
**9**	**2**	Pt/C	Ind	100	0	0	100
**10**		Pd/C	Ind	100	0	0	100
**11**		Pt/C	Thq	100	0	60	40
**12**		Pd/C	Thq	70	65	5	0
**13**		Pt/C	(^i^Pr)_2_NH	100	0	61	39
**14**		Pd/C	(^i^Pr)_2_NH	8	7	1	0
**15** ^c^		Pt/C	(^i^Pr)_2_NEt	100	0	18	82
**16**		Pd/C	(^i^Pr)_2_NEt	33	31	2	0
**17**	**3**	Pt/C	Ind	100	0	0	100
**18**		Pd/C	Ind	23	23	0	0
**19**		Pt/C	Thq	65	57	6	2
**20**		Pd/C	Thq	25	25	0	0
**21**		Pt/C	(^i^Pr)_2_NH	100	2	12	86
**22**		Pd/C	(^i^Pr)_2_NH	6	6	0	0
**23**		Pt/C	(^i^Pr)_2_NEt	55	44	11	0
**24**		Pd/C	(^i^Pr)_2_NEt	14	14	0	0

a Reaction conditions: substrate (0.5 mmol), amine (2.2 mmol), catalyst Pd/C (10 wt %, 0.05 mmol Pd) or Pt/C (10 wt %, 0.05 mmol Pt), *p*-xylene (1.5 mL), 140 °C, 5 h, under Ar and atmospheric pressure.

b Product distribution was determined by GC­(-MS) analysis with internal standard and reference materials (for the detailed procedures, see the SI).

c Data sets were used for comparison, and they were published previously.^4^

All catalyst systems followed a similar reaction pathway: C≡C → C=C → C–C, with the thermodynamically less stable *(Z)*-alkene (products **1a-(** ***Z*** **)** to **3a-(** ***Z*** **)**) consistently emerging as the dominant olefin (Table 2 and Figure 2). For substrate **1**, complete conversion to the fully hydrogenated product (**1b**) was achieved under several conditions (Table 2).

**Figure 2:**
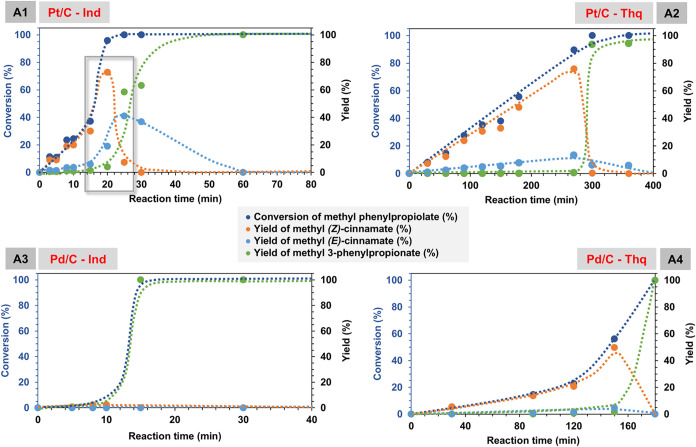
Hydrogen transfer reaction of substrate **1** on Pt/C and Pd/C in *p*-xylene, at 140 °C, under inert conditions (the dashed lines serve as a guide to the eye).

Among the secondary amines, indoline (Ind) and tetrahydroquinoline (Thq) proved to be the most active hydrogen donors with both Pt/C (Table 2, entries 1, 3, 5) and Pd/C (Table 2, entries 2, 4, 6). Pd/C–(^i^Pr)_2_NH showed only marginal catalytic activity (3% conversion, entry 6 in Table 2). Interestingly, Pt/C – (^i^Pr)_2_NH (entry 5 in Table 2) led to full conversion, but with a predominant formation of the *(E)*-alkene (**1a-(** ***E*** **)**), suggesting a much slower second hydrogenation step and a shift in olefin stereoselectivity toward the thermodynamically favored *(E)*-isomer.

Pt/C-(^i^Pr)_2_NEt gave a reaction profile similar to the cyclic secondary amines (entry 7 in Table 2), while Pd/C-(^i^Pr)_2_NEt exhibited only minimal conversion (7%, entry 8 in Table 2). The overall reaction kinetics for the four main systems, including Pt/C–Ind (**A1**), Pt/C–Thq (**A2**), Pd/C–Ind (**A3**), and Pd/C–Thq (**A4**), are illustrated in Figure 2. Pt/C–Ind and Pd/C–Ind display rapid S-like shaped conversion profiles for product **1b**, while Pt/C–Thq and Pd/C–Thq exhibit a more linear progression during the early reaction phase (Figure 2).

A notable case arises for substrate **1** with the Pd/C–Ind system (Figure 2, **A3**): after an initial lag phase with slow formation of both *(Z)-* and *(E)*-isomers, a rapid hydrogenation burst occurs, yielding full conversion to **1b** (Figure 2 **A3**), producing the characteristic S-shaped curve (note: using Pd/C–Ind, due to fast rates to alkene isomers, the yield of olefins are low in the initial regime of the reaction; only the formation of product **1b** is detectable, at 15 min complete conversion to product 1b was observed at 140 °C). In comparison, Pt/C–Ind shows faster olefin formation, favoring the *(Z)*-isomer (73% yield of **1a-(** ***Z*** **)** at 20 min, Figure 2 **A1**), followed by a similarly rapid transition to **1b**. Additionally, a mild *(Z)-*to *(E)*-isomerization for substrate **1** is evident in the Pt/C–Ind system (gray-marked region, Figure 2 **A1**).

The fastest reaction was observed for substrate **2**, with complete conversion achieved within 5 min at 140 °C on Pt/C using Ind as the hydrogen donor (Figure 3: Pt/C–Ind (**B1**), Pt/C–Thq (**B2**), Pd/C–Ind (**B3**), and Pd/C–Thq (**B4**)). When Ind was used as the reducing agent, both Pt/C and Pd/C exhibited high activity, resulting in full conversion to the alkane product (**2b**) (Table 2, entries 9 and 10).

**Figure 3:**
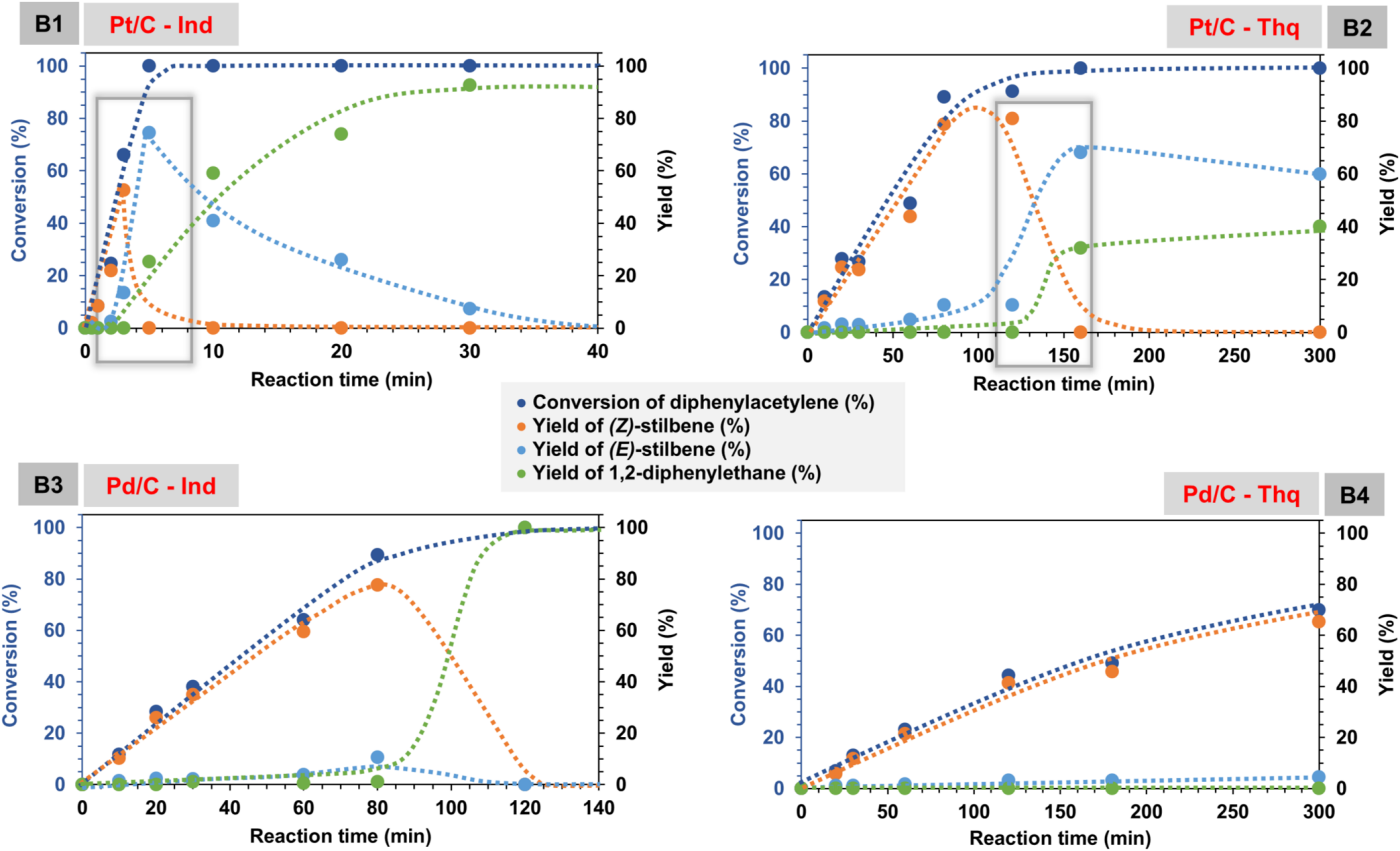
Hydrogen transfer reaction of substrate **2** on Pt/C and Pd/C in *p*-xylene, at 140 °C, under inert conditions (the dashed lines serve as a guide to the eye).

Among the model alkynes, substrate **2** showed the highest metal sensitivity. With Thq as the H-donor, significant differences emerged between the two catalysts (Table 2, entries 11 and 12).

Pd/C afforded moderate conversion (70%), yielding primarily the *(Z)*-alkene formation (**2a-(** ***Z*** **)**) with no overhydrogenation. In contrast, Pt/C favored *(Z)*-alkene formation (Table 2, entry 11, **2a-(*E*)**, 60% yield) and showed significant conversion to the alkane (**2b**, 40%) after 5 h (Table 2, entry 11).

Ind was the most effective H-donor overall. For both Pt/C and Pd/C, full conversion to saturated product **2b** was observed (Table 2, entries 9 and 10). Compared to our previous results^4^ using tertiary alkyl amines, (^i^Pr)_2_NEt yielded moderate conversion on Pd/C (33%), predominantly forming the *(Z)*-alkene, while on Pt/C, it selectively formed **2b** with 86% yield after 5 h.

The noncyclic secondary amine (^i^Pr)_2_NH gave results similar to those of the tertiary amine (^i^Pr)_2_NEt. On Pt/C, 39% of the overhydrogenated **2b** product was formed, with 61% of the *(E)-*alkene (**2a-(** ***E*** **)**) remaining unconverted after 5 h. On Pd/C, only trace amounts of product were detected (**2a-(** ***Z*** **)**, 7%; **2a-(** ***E*** **)**, 1%) (Table 2, entries 13 and 14).

For the Pt/C–Ind system, the highest reaction rate for substrate **2** was observed already at 110 °C, (5.75 × 10^–6^ mol g_cat_^–1^ s^–1^; Table 3). At 140 °C, complete conversion occurred within 5 min. The reaction proceeded through rapid formation of **2a-(** ***Z*** **)** (75% at 5 min), then isomerized to **2a-(** ***E*** **)**, followed by overhydrogenation to **2b** (25% yield at 5 min) (Figure 3 **B1**, gray-marked regime). Notably, the *(Z)-*to *(E)*-isomerization under hydrogen transfer conditions was not observed previously with tertiary amines.^4^ A similar three-step conversion of substrate **2** (HT reaction to **2a-(** ***Z*** **)** followed by isomerization to **2a-(** ***E*** **)**, and final HT to **2b**) was observed using the Pt/C–Thq system (Figure 3 **B2**, gray-marked region). (Note: the internal isomerization of substrate **2** with secondary amines is a topic of a separate study.)

**Table 3:** Activation Parameters (*E*_a_, Δ*H* ^‡^, Δ*S* ^‡^), Initial Rates and TOF Values of the Hydrogen Transfer Reaction of Substrate **1**-**3** on Pt/C and Pd/C in the Presence of Secondary Amines as H-Donors

**substrate** ^a^	**catalyst**	**amine**	***E***_**a**_ **(kJ mol** ^–**1**^ **)** ^b^	**Δ*H*** ^**‡**^ **(kJ mol** ^–**1**^ **)** ^c^	**Δ*S*** ^**‡**^ **(J mol** ^–**1**^ **K** ^**1**^ **)** ^c^	**rate**(mol g_**cat**_^–**1**^ **s** ^–**1**^) at 140 °C^d^	**TOF (s** ^–**1**^ **) at 140 °C** ^e^
**1**	Pt/C	Ind	53 (±5)	50 (±5)	–156 (±13)	2.44 × 10^–6^	2.28 × 10^–2^
	Pd/C	Ind	52 (±3)	49 (±6)	–179 (±16)	3.85 × 10^–7^	2.48 × 10^–3^
	Pt/C	Thq	41 (±6)	37 (±3)	–209 (±9)	2.15 × 10^–7^	2.01 × 10^–3^
	Pd/C	Thq	74 (±8)	70 (±8)	–129 (±21)	2.81 × 10^–7^	1.81 × 10^–3^
for comparison	Pt/C	(^i^Pr)_2_NEt	78 (±2)	74 (±2)	–112 (±6)	7.69 × 10^–7^	4.86 × 10^–3^
**2**	Pt/C	Ind	45 (±4)	42 (±4)	–164 (±12)	5.75 × 10^–6^ (at 110 °C)	3.63 × 10^–2^ (at 110 °C)
	Pt/C	Thq	49 (±3)	46 (±3)	–172 (±6)	2.36 × 10^–6^	1.50 × 10^–2^
**3**	Pt/C	Ind	35 (±2)	32 (±2)	–199 (±6)	5.57 × 10^–6^	3.52 × 10^–2^
	Pt/C	Thq	52 (±3)	49 (±3)	–170 (±9)	1.09 × 10^–6^	6.92 × 10^–3^

a Site density is based on H_2_ chemisorption (see the SI). Reaction conditions: substrates **1**–**3** (0.5 mmol), amines (2.2 mmol), catalysts Pd/C (10 wt %, 0.05 mmol Pd) or Pt/C (10 wt %, 0.05 mmol Pt), *p*-xylene (1.5 mL), at a given temperature, under Ar and atmospheric pressure.

b For detailed kinetic measurements, see SI, Tables S1 **–** S10. Activation energy was calculated using the Arrhenius equation.

c Enthalpy and entropy values were calculated based on the Eyring equation (see the SI, Scheme S1, Table S10). Error bars were calculated based on the LINEST method (the linear least-squares method).

d The initial reaction rate was deduced from the slope of the linear fit to the conversion versus reaction time plot in the linear region at low conversion.

e TOF values were determined from rates normalized to accessible metal sites and calculated in the unit of (mol mol_(surf.metal)_^–1^ s^–1^) and shortened as (s^–1^).

Using 1-phenyl-1-propyne (substrate **3**) as a model alkyne, independently of the type of the amine partner, the results obtained with Pt/C always exceeded those measured with Pd/C (Table 2, entries 17–24, Figure 4). The cyclic secondary amines gave the highest measured yield for the *(Z)*-isomer (product **3a-(** ***Z*** **)**, Table 2) of 23% using Ind with Pd/C and 57% using Thq with Pt/C, while the alkyl secondary amine ((^i^Pr)_2_NH) gave the lowest conversion and yield, 6% for **3a-(** ***Z*** **)** (Table 2, entry 22).

**Figure 4:**
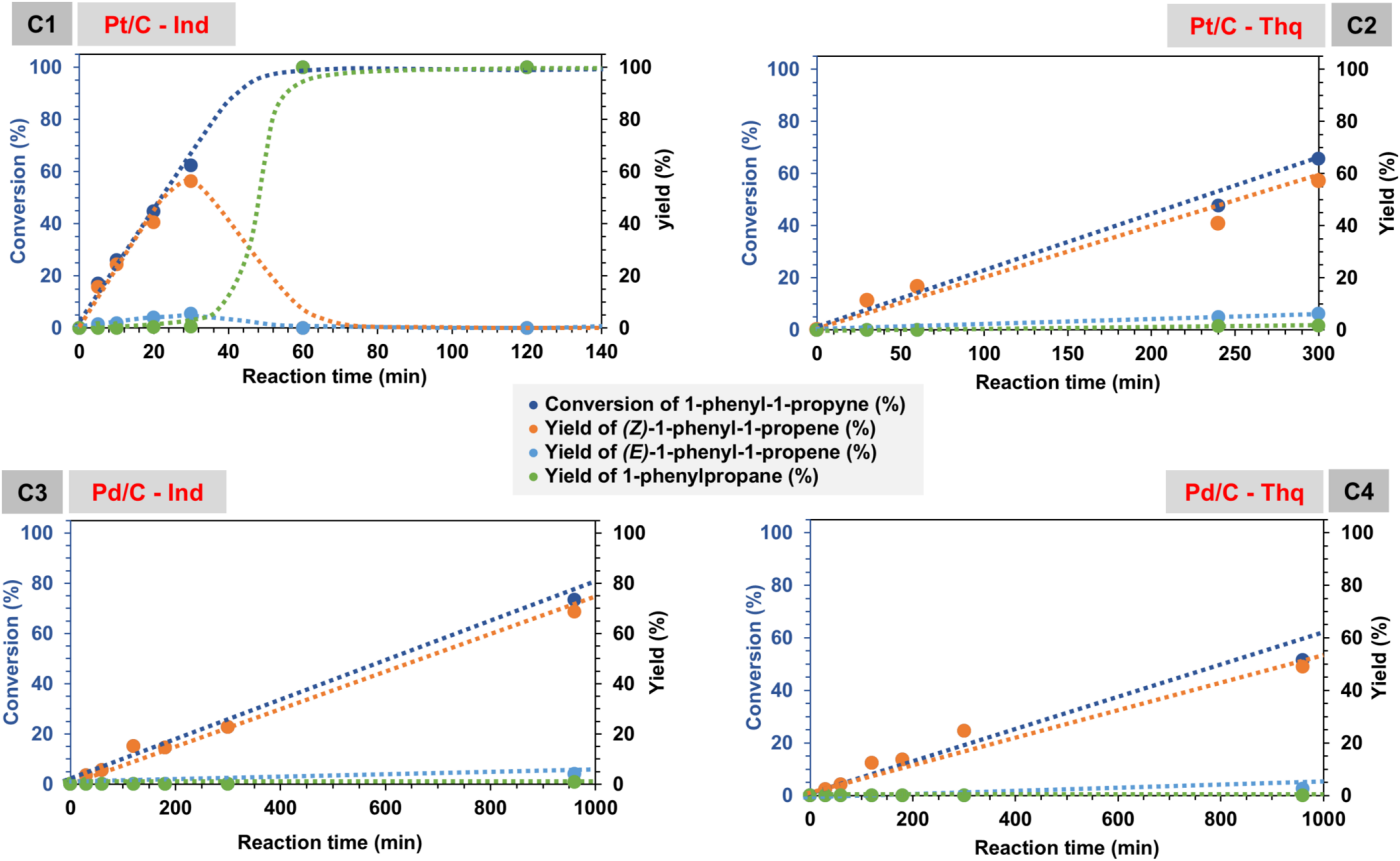
Hydrogen transfer reaction of substrate **3** on Pt/C and Pd/C in *p*-xylene, at 140 °C, under inert conditions (the dashed lines serve as a guide to the eye).

On Pt/C with Ind, complete conversion to product **3b** was achieved, whereas with Thq, 65% conversion was observed, with **3a-(** ***Z*** **)** as the dominant intermediate (57% yield; Table 2, entries 19 and 20). The reaction profiles for Pt/C–Ind and Pt/C–Thq exhibit distinct kinetics. In the presence of Ind, rapid formation of the *(Z)*-alkene **3a-(** ***Z*** **)** was followed by complete hydrogenation to **3b** (Table 2 entry 17; Figure 4). In contrast, with Thq as the hydrogen donor, the *(Z)*-isomer accumulated in the early phase of the reaction, followed by a gradual, sequential conversion to **3b**, reaching completion only after 16 h (Figure 4 **C2**) (Note: at 16 h reaction time full conversion was measured with the distribution of 9% yield /**3a-(** ***E*** **)** and 91% yield of **3b**). The highest measured yield and selectivity toward the *(Z)*-isomers, dependent on the catalyst system, are summarized in Table 4.

**Table 4:** Highest Measured Yields and Selectivity Towards the (*Z*)-Isomers (**1a-(** ***Z*** **)**–**3a-(** ***Z*** **)**) on the Most Active Catalyst Systems from the Graphical Summary in Figures 2, 3, and 4 Including the (*E*)-Isomers (**1a-(** ***E*** **)–3a-(** ***E*** **)**) and Overhydrogenated (**1b**–**3b**) Products

			**catalyst system ([M]/C**–**amine)**			
			**Pt/C**–**Ind**	**Pt/C**–**Thq**	**Pd/C**–**Ind**	**Pd/C**–**Thq**
			**corresponding yield (%) and selectivity [%]**			
substrate^a^	**reaction time (min)**	**conversion (%)**	**(** ***Z*** **)/(** ***E***)/**b** product	**(** ***Z*** **)/(** ***E***)/**b** product	**(** ***Z*** **)/(** ***E***)/**b** product	**(** ***Z*** **)/(** ***E***)/**b** product
**1**	20	96	(73/20/4) [75/21/4]	-	-	-
	270	90	-	(76/13/1) [84/14/2]	-	-
	15	100	-	-	(0/0/100)^b^ [0/0/100]	-
	150	56	-	-	-	(50/5/1) [89/9/2]
**2**	3	66	(53/13/0) [80/20/0]	-	-	-
	120	91	-	(81/10/0) [90/10/0]	-	-
	80	89	-	-	(78/11/0) [88/12/0]	-
	300	70	-	-	-	(66/4/0) [94/6/0]
**3**	30	62	(56/5/1) [90/8/2]	-	-	-
	300	65	-	(57/6/2) [88/9/2]	-	-
	960	74	-	-	(69/4/1) [93/5/2]	-
	960	52	-	-	-	(50/2/0) [96/4/0]

a Reaction conditions: substrate **1**–**3** (0.5 mmol), amines (2.2 mmol), catalyst Pd/C (10 wt %, 0.05 mmol Pd) or Pt/C (10 wt %, 0.05 mmol Pt), *p*-xylene (1.5 mL), at 140 °C, under Ar and atmospheric pressure.

b Due to the rapid conversion to product **1b** even at lower temperature (please see SI), the corresponding interim product distribution at higher conversion was not determined.

To further elucidate the catalytic activity and selectivity trends, we monitored the hydrogen transfer reactions across all three model substrates. The most active systems, including Pt/C–Ind, Pt/C–Thq, Pd/C–Ind, and Pd/C–Thq, were selected for kinetic evaluation, and the corresponding parameters were determined (Table 3, Figures 5 and 6). These four systems consistently demonstrated the highest reaction rates across the substrate set.

**Figure 5:**
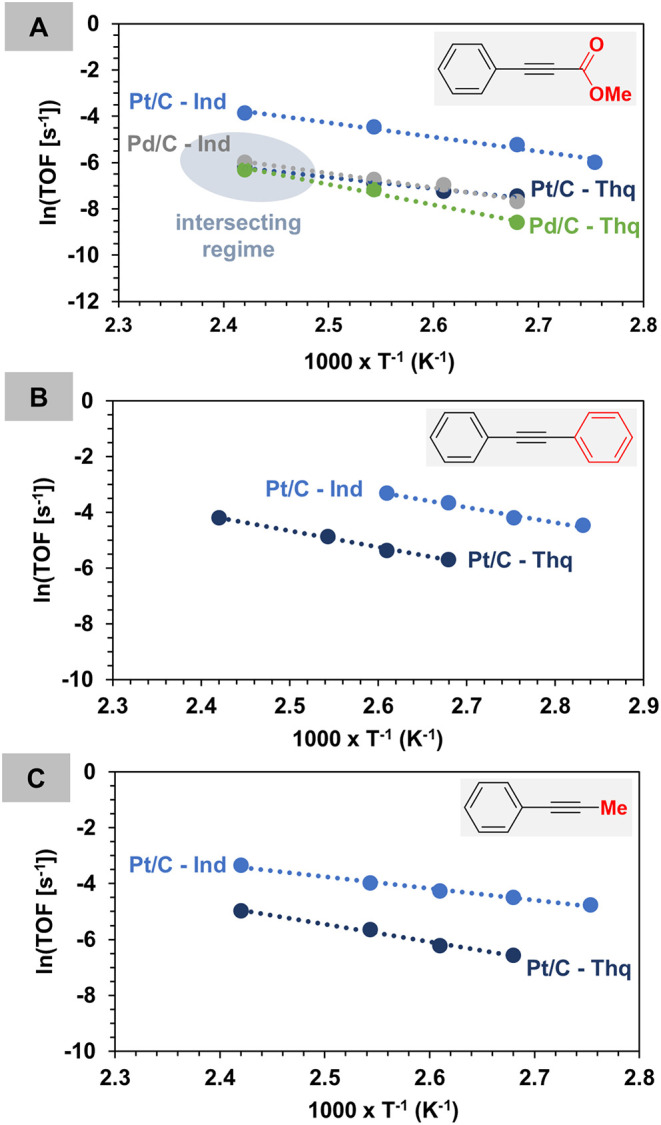
Arrhenius plots of the corresponding catalyst systems using substrate **1** (part A), substrate **2** (part B), and substrate **3** (part C).

**Figure 6:**
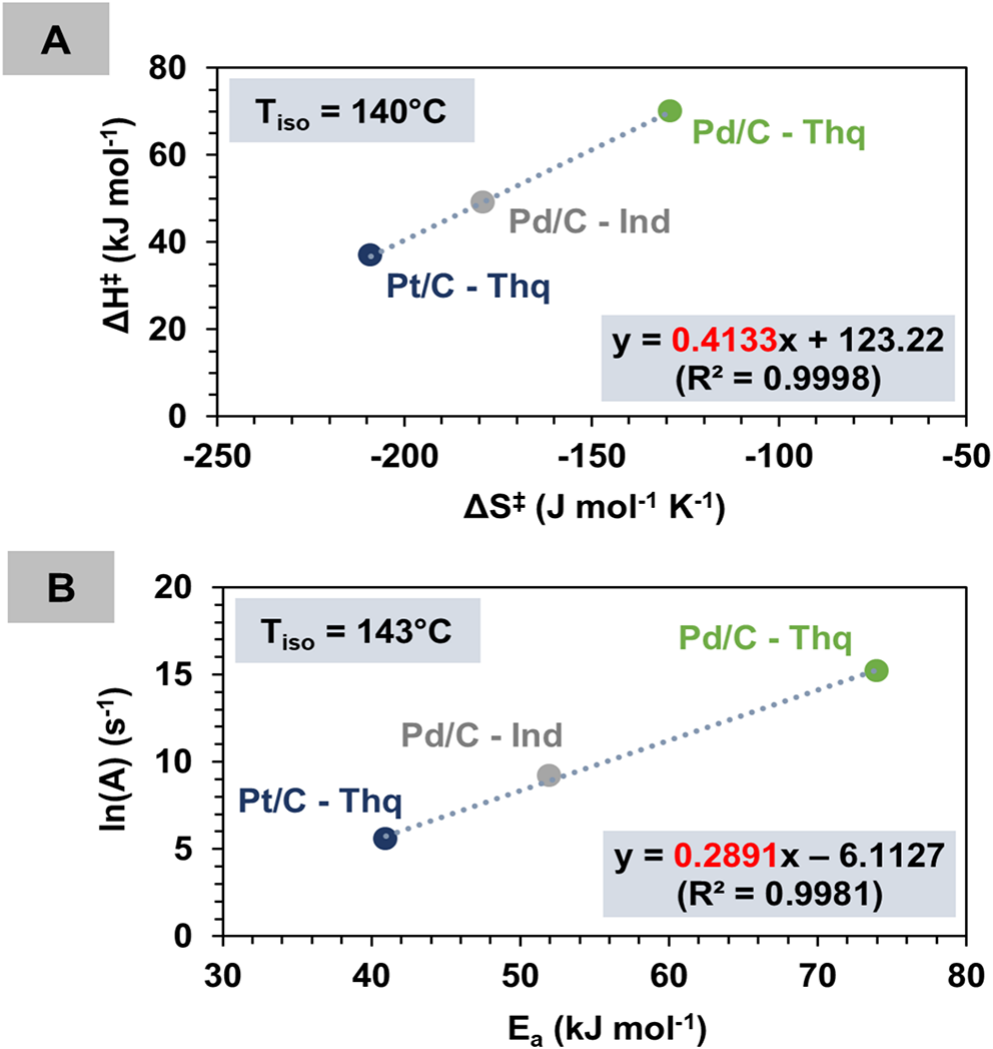
Compensation effect for substrate **1** on Pt/C–Thq, Pd/C–Ind and Pd/C–Thq catalyst systems. (A) Δ*H* ^‡^ vs Δ*S* ^‡^ correlation. (B) Constable plot. T_iso_ values were calculated from the slope of the Δ*H* ^‡^ vs Δ*S* ^‡^ plot (A) and using *T*_iso_ = (*R* × slope)^−1^ × 1000 (in K) from ln­(*A*) vs *E*_a_ correlation (B). ln­(*A*) values were calculated from the slope of the Arrhenius plots, and *k*_iso_ values were calculated from the equations: ln­(*A*) = *b* × *E*_a_ + *c*; *b* = (*R* × *T*_iso_)^−1^ and *c* = ln­(*k*_iso_).^25–32^ .

For substrate **1**, Pt/C and Pd/C with Ind showed nearly identical activation parameters: the activation energy (*E*_a_) was 52–53 kJ mol^–1^, and the activation enthalpy (Δ*H* ^‡^) was 49–50 kJ mol^–1^ for both catalysts. The activation entropy (Δ*S* ^‡^) was −156 J mol^–1^ K^–1^ for Pt/C and slightly more negative for Pd/C at −179 J mol^–1^ K^–1^ (Table 3). When Thq was used as the H-donor, higher activation parameters were observed on Pd/C (*E*_a_ = 74 kJ mol^–1^, Δ*H* ^‡^ = 70 kJ mol^–1^), while the activation entropy was less negative (−129 J mol^–1^ K^–1^) compared to Pt/C (−209 J mol^–1^ K^–1^) (Table 3).

For substrate **3**, the simplest internal alkyne in our series, we compared the two fastest and most selective catalytic systems: Pt/C–Ind and Pt/C–Thq (Table 3). The lowest activation energy was measured on Pt/C–Ind (*E*_a_ = 35 kJ mol^–1^), accompanied by a correspondingly low Δ*H* ^‡^. The activation entropy for this system was −199 J mol^–1^ K^–1^, more negative than for Pt/C–Thq (−170 J mol^–1^ K^–1^), indicating greater entropic stabilization of the transition state when Ind is used as the H-donor.

The least negative activation entropy (−129 J mol^–1^ K^–1^) was recorded for substrate **1** on Pd/C–Thq using a cyclic secondary amine. For comparison, the closest Δ*S* ^‡^ value among tertiary alkyl amines was observed with (^i^Pr)_2_NEt (−112 J mol^–1^ K^–1^; Table 3), suggesting that secondary amines can modulate the activation entropy more effectively than their tertiary counterparts.

Regardless of the substituent pattern on the C≡C triple bond, all catalyst systems yielded highly negative activation entropies. This strongly negative regime suggests that the transition state involves a highly ordered assembly of reacting species, indicating a significant reduction in degrees of freedom along the reaction coordinate. Activation energies were calculated using the Arrhenius eq (Table 3, Tables S1–S9).

A particularly notable observation arises for substrate **1**, where three Arrhenius plots intersect at a common point (Figure 6A). This intersection indicates the presence of an enthalpy–entropy compensation effect.^25–41^ Interestingly, this phenomenon was not observed for substrates **2** or **3** in the current study. This stands in contrast to our previous work with tertiary alkyl amines,^2^ ^a^ where substrates **2** and **3** exhibited linear compensation behavior across the same catalyst system. Here, however, substrate **1** displays a linear correlation across three distinct catalyst systems, including Pt/C–Thq, Pd/C–Ind, and Pd/C–Thq, highlighting the substrate-dependent nature of this compensatory behavior (Figure 6 and Figure 6A).

The compensation effect, first reported by Wilson in 1908 in the context of electron emission from heated platinum in a hydrogen atmosphere,^31,33^ has since been studied extensively, especially in heterogeneous catalysis.^34–37^ Often termed the *compensation law* ^31,38^ or *isokinetic relationship*, it describes a linear correlation between ln­(*A*) and *E*_a_ [ln­(*A*) = *b* × *E*_a_ + *c*], where *A* is the pre-exponential factor and *E*_a_ is the apparent activation energy. This relationship defines an isokinetic temperature (*T*_iso_) at which all reactions in the set proceed at the same rate constant. While *T*_*i*so_ typically lies outside the temperature ranges of experimentally achievable reaction conditions, exceptions exist.^31,39^

In the current system, *T*_iso_ falls within the reaction experimental regime for both Pt/C and Pd/C, allowing direct comparison with measured reaction rates. From the ln­(A) vs *E*_a_ plot (Figure 6B), *T*_iso_ was calculated as 143 °C with a corresponding rate constant *k*_iso_ = 2.25 × 10^–3^ (±0.3) s^–1^. A nearly identical *T*_iso_ of 140 °C was derived from the Δ*H* ^‡^ vs Δ*S* ^‡^ correlation (Figure 6A), yielding *k*_iso_ = 2.00 × 10^–3^ (±0.3) s^–1^. These findings offer compelling support for a unified enthalpy–entropy compensation pathway across the examined catalysts. To rationalize the observed activity and selectivity trends, a mechanistic hypothesis was proposed (Figure 7), supported by our DFT calculations.

**Figure 7:**
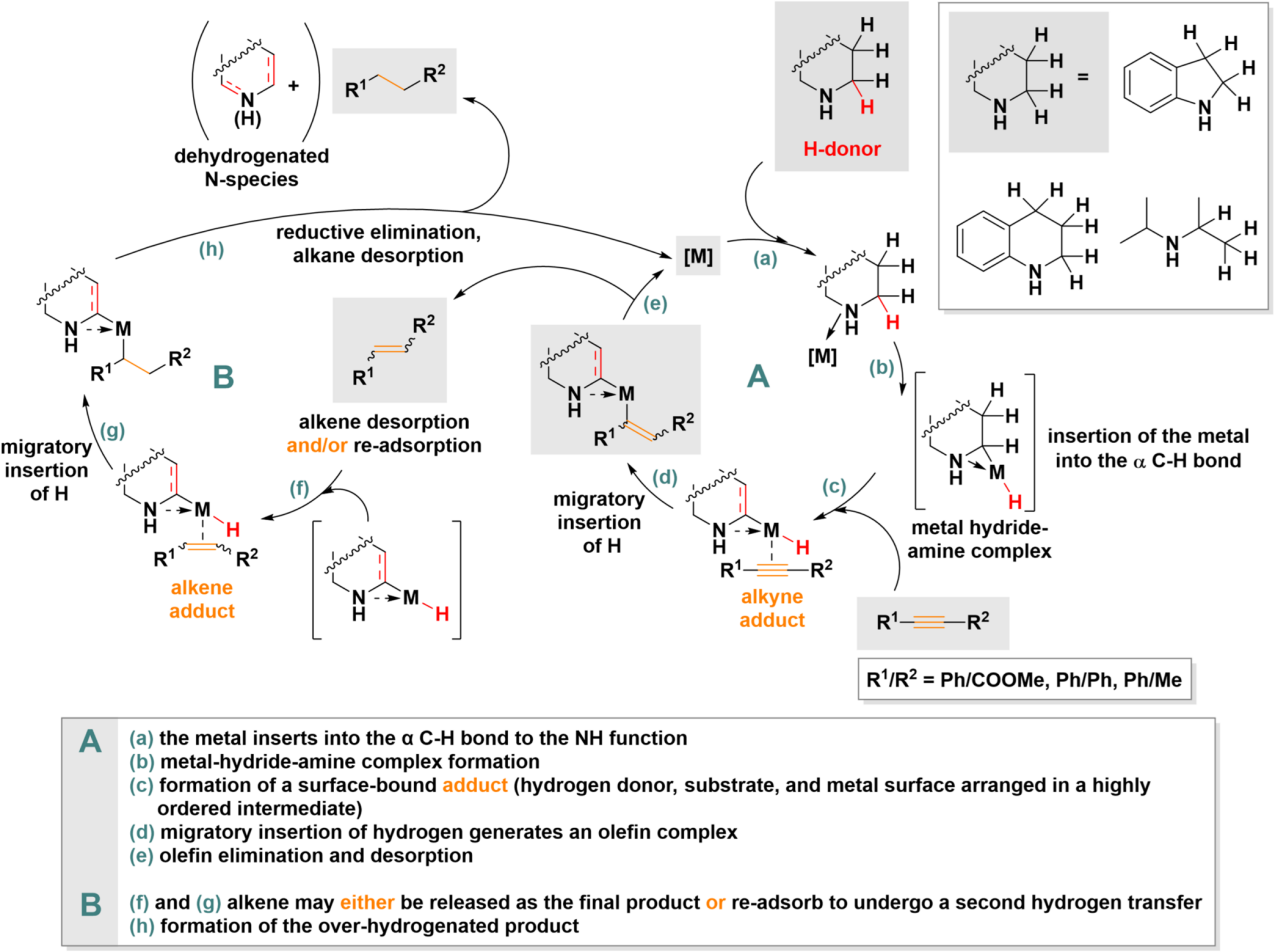
Proposed reaction mechanism for catalytic hydrogen transfer to internal alkynes in the presence of secondary amines as H-donors on carbon-supported noble metals, the alkyne (cycle **A**) and the alkene cycle (cycle **B**).

Secondary amines served as hydrogen donors, resulting in the formation of enamines or other unsaturated nitrogen-containing species. We propose a mechanism in which the metal inserts into the C–H bond α to the NH function (Figure 7, step **a**), forming a metal-hydride-amine complex (Figure 7, step **b**). This is followed by the formation of a surface-bound adduct (Figure 7, step **c**), consisting of the hydrogen donor, substrate, and metal surface arranged in a highly ordered intermediate. Migratory insertion of hydrogen generates an olefin complex (Figure 7, step **d**), and subsequently, olefin elimination and desorption (Figure 7, step **e**) complete the catalytic cycle. The resulting alkene may either be released as the final product or readsorb to undergo a second hydrogen transfer (Figure 7, step **f** and **g**), ultimately yielding the fully saturated product (Figure 7, step **h**).

The consistently large, negative activation entropies observed across all tested catalyst systems support a concerted reaction pathway, as indicated by the calculated activation parameters, despite the entropy penalty. Furthermore, experimental evidence shows that hydrogen transfer proceeds sequentially, consistent with a highly ordered transformation pathway that is unaffected by the steric bulk of the alkynes.

Tertiary alkyl amines form iminium ion complexes upon hydrogen donation,^20–24^ whereas secondary amines retain NH bonds during activation, typically generating imine-hydride metal complexes.^20–24^ Notably, secondary amines preferentially form stable metal complexes rather than dissociating into free [M–H] and enamine species,^23,24^ further supporting the proposed surface-bound alkyne adduct intermediate and explaining the consistently negative activation entropy values (Table 3). For instance, the entropy of activation for (^i^Pr)_2_NEt with substrate **1** on Pt/C was calculated as −112 J mol^–1^ K^–1^, aligning with previously reported systems,^4^ but significantly less negative than those observed for secondary cyclic amines (Table 3).

In order to describe the reusability of the studied catalyst system, recycling experiments were carried out. HT reaction of substrate **2** with Pt/C – (^i^Pr)_2_NH was measured in 4 consecutive catalytic runs (Figure 8) under inert conditions (for details, please see SI). Each run was carried out with complete conversion and with the following product distribution in yield (%) after 5 h, **1a-(** ***E*** **)/2b**: run-1/61:39 (Table 2, entry 13), run-2/57:43, run-3/63:37, run-4/62:38. This trend shows clearly the reusability of the catalyst system, and due to the minimal change in the particle size distribution (before reaction: 3.6 (±2.7) nm (Table 1, Figure S2), after reaction 3.2 (±1.4) nm (Figure S2)) after the reaction, it can be concluded that the catalyst is stable, no major morphological change was observed. We selected the Pt/C – (^i^Pr)_2_NH catalyst system to demonstrate that even after multiple usage, it kept its catalytic activity toward all transformations, including the unique interim isomerization to the *(E)*-olefin.

**Figure 8:**
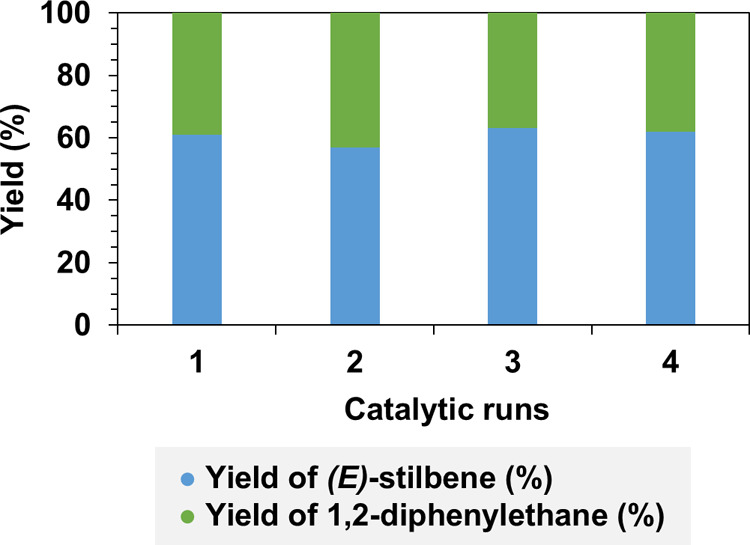
Recycling experiments using Pt/C – (^i^Pr)_2_NH catalyst system for the HT reaction and internal isomerization of substrate **2** in *p*-xylene at 140 °C after 5 h reaction time.

The reaction order for substrates **1**–**3** was evaluated on representative catalysts (see SI, Figures S4–S9, Tables S11–S16): substrate **1** on Pt/C-Ind, Pd/C-Ind, Pt/C-Thq, and Pd/C-Thq; substrate **2** on Pt/C-Ind; and substrate **3** on Pt/C-Ind. In all cases, zero-order kinetics with respect to the substrate were observed. Given the excess of amine donors (substrate/amine ratio = 1:4.4), no dependence of the reaction rate on amine concentration was found. Control experiments confirmed that no reaction occurred in the absence of an amine donor. Similarly, no conversion was observed without a metal catalyst, confirming that hydrogen transfer is strictly metal-catalyzed.

Parallel with the initial reaction rate of olefins (calculated from the conversion values), we determined the formation rate (calculated from the yield of oxidized amine) of the oxidized Ind (1*H*-indoline/HInd) and oxidized Thq (quinoline/Quin) (Table S17) using Pt/C. The formation rate of HInd and Quin was in general slower (in case of substrate **1** the values were highly comparable, Table S17 entry 1–2): using Thq in case of substrate **2** it was 1 order of magnitude slower and for substrate **3** the formation rate of Quin was 2 orders of magnitude slower than the olefin formation rate (Table S17, entry 4 and entry 6), respectively. Furthermore, these differences in the measured values (Table S17) indicate the presence of a surface adduct (Figure 7) rather than a sequential hydrogen shift from a [M–H] surface-linked species.

The question arises as to whether molecular hydrogen is formed under the conditions of the studied reaction and, if so, what the status of gaseous hydrogen is. In order to get insight into this issue, we carried out the following cross-experiments (Tables S18 and S19): we sampled the HT reaction of substrate **1** with Ind as H-donor after 5 min and 1 h reaction time at 140 °C, using Pt/C, and another experiment under the same reaction conditions without the addition of the substrate after 5 min. These experiments showed that there is only a minimal presence of H_2_ in the gas phase (Table S18), 0.0–2.1% of the calculated maximum available H amount from the amine).

### 2.2 Computational Details

Due to the experimental challenges of *in situ* spectroscopic analysis of surface-bound species, we performed DFT calculations to further investigate the observed trends (Figures 9–12, Tables 5–6).^42–57^ The computational results were consistent with experimental findings, showing that electron distribution within the substrates critically influences both reactivity and binding strength to the catalytic surface (Figure 9). Full computational details are provided in the Supporting Information (Tables S20–S25, Figures S10–S21).

**Figure 9:**
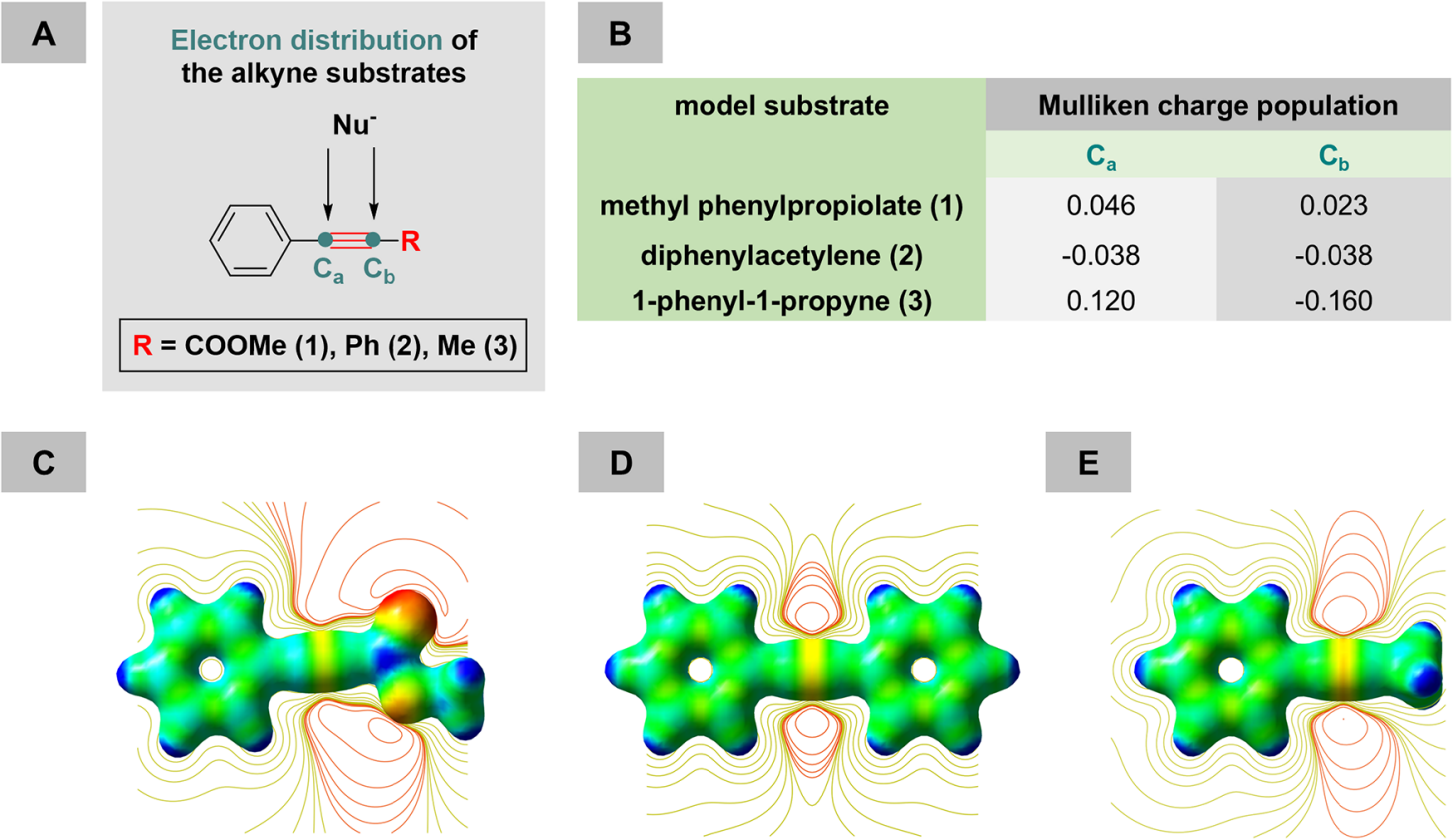
Electronic structure properties of the substrates characterized by Mulliken population analysis and electrostatic potential mapping. (A) Schematic representation of the substrate molecule, with alkynyl-carbon atoms labeled as nucleophilic centers. (B) Determined Mulliken charges of the respective active carbon atoms across different substrates. (C–E) Electrostatic potential mapping onto isosurfaces of the total electron density for substrates **1**–**3**, plotted at a contour level of 0.05 a.u., and color-mapped according to the electrostatic potential (ESP) at each point on the surface. The ESP potential color ranges from −0.05 a.u. (electron-rich regions) to 0.3 a.u. (electron-deficient regions), with equipotential lines superimposed.

**Table 5:** Binding Energies of the Model Substrates and Intermediates on Pt/C and Pd/C

	**binding energy (eV)**	
**model substrate**	**Pt(111)**	**Pd(111)**
**methyl phenylpropiolate (1)**	–3.80	–3.82
**1a-(** ***Z*** **)**	–3.38	–3.47
**1a-(** ***E*** **)**	–3.61	–3.62
**diphenylacetylene (2)**	–4.33	–4.64
**2a-(** ***Z*** **)**	–3.79	–4.30
**2a-(** ***E*** **)**	–4.63	–4.91
**1-phenyl-1-propyne (3)**	–3.60	–3.62
**3a-(** ***Z*** **)**	–3.19	–3.19
**3a-(** ***E*** **)**	–3.64	–3.32

**Table 6:** Binding Energies of the Used Amines, Observed and Possible Intermediates on Pt/C and Pd/C

	**binding energy (eV)**	
**adsorbate**	**Pt(111)**	**Pd(111)**
**Ind**	–3.13	–2.97
**HInd**	–3.07	–3.00
**Thq**	–3.08	–3.02
**Quin**	–3.29	–3.47
**1,2-Di**	–3.85	–3.70
**3,4-Di**	–3.19	–3.17

The theoretical investigation revealed that the reactivity of the alkynes is closely related to the electron distribution at the reactive triple bond. Specifically, for substrate **2** (diphenylacetylene), Mulliken charge analysis revealed equal negative charges on both alkyne carbon atoms (−0.038 on C_a_ and −0.038 on C_b_, Figure 9B), indicating a highly nucleophilic triple bond favorable for electrophilic hydrogen addition. This is supported by high contour line density in the p-bond region (Figure 9C–E), highlighting a concentrated, accessible electron density suitable for nucleophilic reactivity (Figure 9C,D,E). Correspondingly, substrate **2** displayed the highest reactivity experimentally, achieving full conversion within 5 min at 140 °C on Pt/C using indoline (Ind) as the hydrogen donor, corroborating the theoretical prediction of enhanced reactivity (Figure 9B).

In contrast, substrate **1** (methyl phenylpropiolate) showed slightly positive charges on the alkyne carbon atoms (0.046 on C_a_ and 0.023 on C_b_, Figure 9B), reflecting an electron-deficient C≡C bond. Contour plots show highly delocalized p-electron density, partly due to electron-withdrawing effects from adjacent oxygen atoms (Figure 9C), leading to reduced nucleophilicity and lower reactivity. These electronic features correlate with the experimental observation of slower reaction rates and less favorable product distribution for substrate **1** compared to substrate **2**.

Substrate **3** exhibits a pronounced asymmetry in charge distribution, with C_a_ bearing a significantly positive charge (+0.12) and C_b_ carrying notable negative charge (−0.16) (Figure 9B). While the negative charge on C_b_ implies potential nucleophilic character, the strong electrostatic asymmetry generates an uneven potential field that likely hinders electrophile approach. This asymmetry also reduces spatial overlap between the regions of high electron densities and the incoming electrophilic hydrogen. This effect is compounded by the more delocalized nature of the p-bond in substrate **3**, evidenced by the lower contour line density along the C≡C axis (Figure 9E). The resulting diffuse electron cloud weakens the nucleophilic character of the triple bond. Together, these steric and electronic factors align with the experimentally observed lower reactivity of substrate **3**.

In assessing the overall reactivity, it is crucial to consider the net electronic character of the entire C≡C unit. Among the three substrates, only substrate **2** exhibits a net negative Mulliken charge across both carbon atoms (−0.076), whereas substrates **1** and **3** are net positive and near-neutral charge, respectively (Figure 9B). This cumulative electron density supports the higher reactivity of substrate **2**, reinforcing the correlation between electronic structure and observed reactivity.

Table 5 summarizes the computed binding energies for the model substrates on the Pt(111) and Pd(111) surfaces. All values were calculated using a 6 × 6 metal slab model with four layers, where the bottom two layers were fixed. We computed the binding energies of all reactants, products, and intermediates on the metal surfaces (Figure 10, Tables 5 and 6, SI Figures S11 and S12).

**Figure 10:**
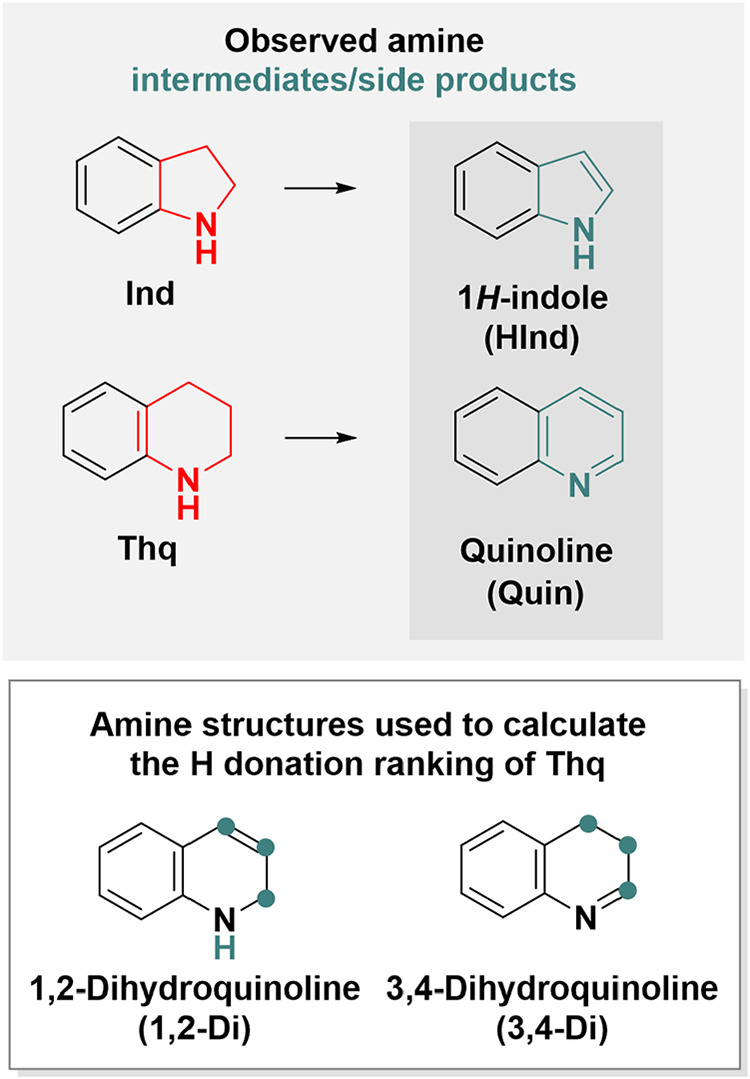
Observed amine intermediates and side products forming during the hydrogen transferring route.

In all cases, the alkyne binds more strongly to the metal than does the corresponding *(Z)*-alkene. This enhanced binding is attributed to the greater electronic overlap between the p orbitals of the alkyne triple bond and the metal d orbitals. These results indicate that the alkyne preferentially adsorbs onto the catalyst surface, which has direct implications for the observed intermediates and products. The relative adsorption energies of reactants, intermediates, and products thereby help rationalize the observed products in the gas phase, as well as provide qualitative insights into relative performance. We note that this can be used to explain the observed products in the gas phase, but not necessarily the full catalytic mechanism and all intermediates occurring on the surface itself. Nevertheless, the relative calculated affinities and culminating competitive adsorption/desorption processes provide clear insight into the experimental observations.

Upon partial hydrogenation, the formation of the *(Z)*-alkene product (with its substantially worse binding affinity) facilitates its desorption, thereby inhibiting further hydrogenation until the surface is largely depleted of alkynes (Table 5). Consequently, the hydrogenation step emerges as the rate-limiting process rather than the initial adsorption event, as produced *(Z)*-alkene is quickly replaced by a more favorably binding unreacted alkyne.

Substrate **2** exhibits the strongest binding affinity among the three, with calculated energies of −4.33 eV on Pt(111) and −4.64 eV on Pd(111) (Table 5). This strong adsorption likely facilitates a higher reaction rate by promoting efficient displacement of the partially hydrogenated product. Notably, for substrates **1** and **2**, the binding energy difference between the alkyne and *(Z)*-alkene is greater on Pt(111) than on Pd(111) (Table 5), supporting the idea that stronger alkyne–surface interactions enhance hydrogenation efficiency by ensuring both effective activation and turnover via desorption and substrate exchange.

Binding energy analysis of the model amines further reinforces the mechanistic insights. As shown in Table 6, the fully dehydrogenated indole derivative (HInd) binds slightly more weakly than both the partially and fully dehydrogenated products of tetrahydroquinoline (Quin) on both Pt(111) and Pd(111). This disparity arises from structural and electronic differences: HInd retains a five-membered ring system that interacts suboptimally with the metal surface, while Quin features a planar, fused double six-membered ring system that aligns more effectively with the metal lattice of Pt or Pd (SI Figures S13 and S14), enhancing adsorption.

These trends extend to those of partially hydrogenated quinolines. Among them, 3,4-dihydroquinoline (3,4-Di) binds most strongly due to its intact six-membered ring engaging the metal surface and a nitrogen atom that remains coordinated. In contrast, 1,2-dihydroquinoline (1,2-Di) binds less strongly than both Quin and 3,4-Di, although it still binds more strongly than the parent Thq, owing to the less favorable adsorption geometry. These variations in binding strength reflect subtle electronic and steric effects that govern desorption dynamics and thus influence the overall catalytic performance (Figure 10).

These results are consistent with experimental observations, where the Ind system consistently demonstrates reaction rates higher than those of Thq. The weaker binding of HInd facilitates the rapid desorption of its hydrogenation products, minimizing surface blockage and sustaining higher catalytic turnover. In contrast, the stronger adsorption of quin leads to prolonged surface residence times, effectively reducing active site availability and impeding further reactivity, characteristic of catalyst poisoning. This effect is even more pronounced with 1,2-Di, whose stronger surface affinity exacerbates poisoning by inhibiting the desorption of the dehydrogenated amine.

Meanwhile, 3,4-Di’s, with intermediate binding strength, does not accumulate significantly on the surface and thus has a limited impact on the overall turnover rate. However, it is not the primary intermediate driving the observed reactivity trends, as the hydrogen at position 2 was calculated to be the first one to detach (Table S20). Together, these theoretical insights support the mechanistic interpretation that small structural differences in the molecular structure of hydrogenated products can significantly influence adsorption behavior and catalytic efficiency. Figure 11 illustrates the DFT-calculated lowest-energy conformers along the reaction coordinate for the initial hydrogen transfer step, leading to either *(Z)*- or *(E)*-alkene products.

**Figure 11:**
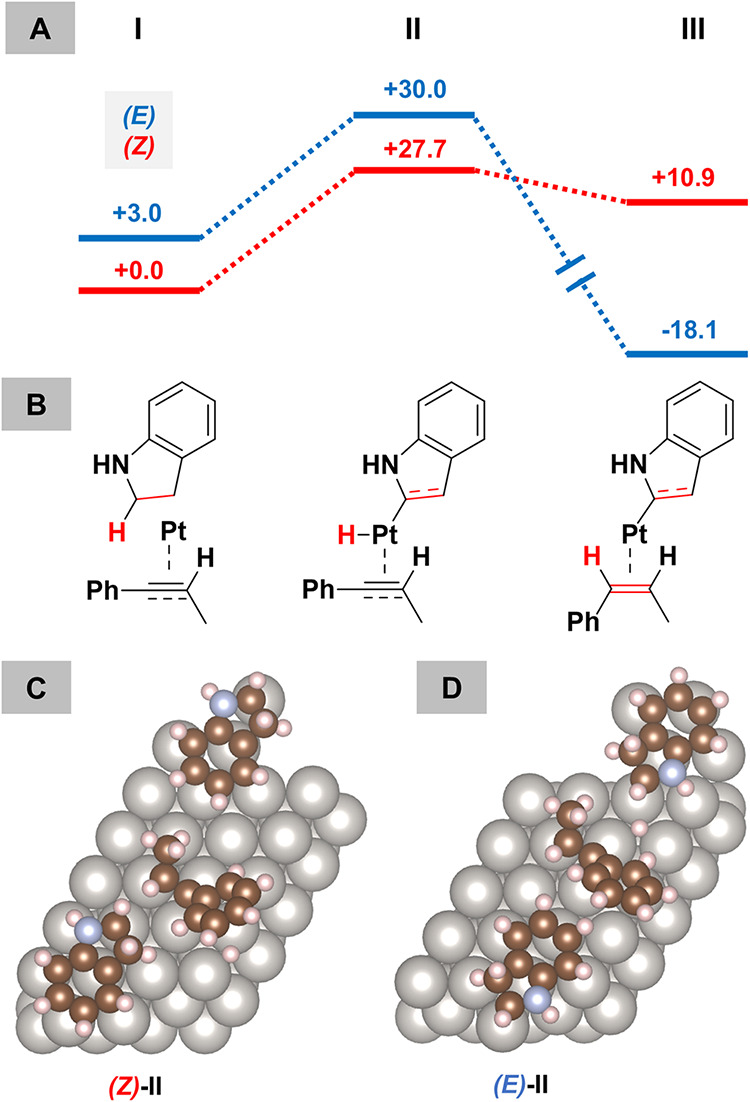
Intermediate state plots representing the DFT-calculated lowest-energy conformers of the first hydrogen transfer process forming the *(Z)*-and *(E)*-alkenes. (A) The intermediate energy plot (values given in kJ mol^–1^) with **I**, starting materials including Ind and alkyne; **II**, hydrogen transfer intermediates; **III**, product states. Notice that the relative distances across the energies are adjusted for clarity, but the relative magnitude was kept. (B) Schematic representation of the reaction process. (C) Lowest-energy *(Z)*-forming conformer. (D) Lowest-energy *(E)*-forming conformer.

In Figure 11A, the energy profile is divided into three key states: **I**, the starting materials (amine and alkyne); **II**, the hydrogen transfer intermediates; and **III**, the final product states. Panels **B** and **C** illustrate the lowest-energy conformers along the *(Z)*- and *(E)*-forming pathways, respectively. In exploring the sequential hydrogenation of substrate **3** with the indoline system, we constructed complex models to simulate high surface coverage conditions, extending beyond the scope of our earlier work (Figures 11 and 12, SI Figures S18, S10C, S10D).^4^ To accommodate the increased steric bulk of the larger amine, a Pt(111) slab with a 4 × 5 unit cell was employed (SI Figure S10C, S10D).

The calculations show that the *(E)*-forming pathway begins at a slightly higher energy in state **I**, indicating less favorable adsorption compared to the *(Z)*-forming pathway. Furthermore, among all modeled hydrogen transfer intermediates, the lowest-energy *(Z)*-forming conformer is 2.3 kJ mol^–1^ more stable than its *(E)*-forming conformer (Figure 11), suggesting a modest thermodynamic preference for the *(Z)* pathway. Interestingly, the product state along the *(Z)*-forming pathway lies higher in energy than the intermediate state **II**, particularly in the Thq system (Figure 10), and to a lesser extent in the Ind system (SI Figure S18). However, this product is only weakly adsorbed, and with a calculated 0.42 eV binding energy preference for the alkyne **1** over product **1a-(** ***Z*** **)**, desorption is expected to occur rapidly, limiting the persistence of any stable intermediate **III**. This leads to a central mechanistic question: does the stabilization of the *(Z)*-isomer primarily originate from electronic factors (Figure 10), or from steric effects (Figures 11 and 12)? To address this, we systematically examined alternative conformations (SI Figures S19–S21), revealing that both effects may contribute, though their relative importance likely varies between systems.

As previously performed for tertiary amines under high surface coverage,^4^ key surface distances were calculated on the Pt(111) using substrate **3** as the model alkyne, with Ind and Thq serving as hydrogen donors. A consistent trend emerged with both amines: in the *(Z)*-**II** conformer, the carbon atom nearest to the Pt surface was found to be significantly farther away, 3.34 Å with Ind and 3.54 Å with Thq (Figure 12A,B, respectively). In contrast, the corresponding distances in the *(E)*-**II** conformer were notably shorter, 2.32 Å with Ind and 2.33 Å with Thq, than those previously found.^4^ These results indicate that the stabilization of the *(Z)*-II intermediate with respect to the *(E)*-II intermediate (Figure 11, SI Figure S18) is due to electronic effects, as the sterics favor the *(E)* intermediate, as was also observed in our previous work.^4^

**Figure 12:**
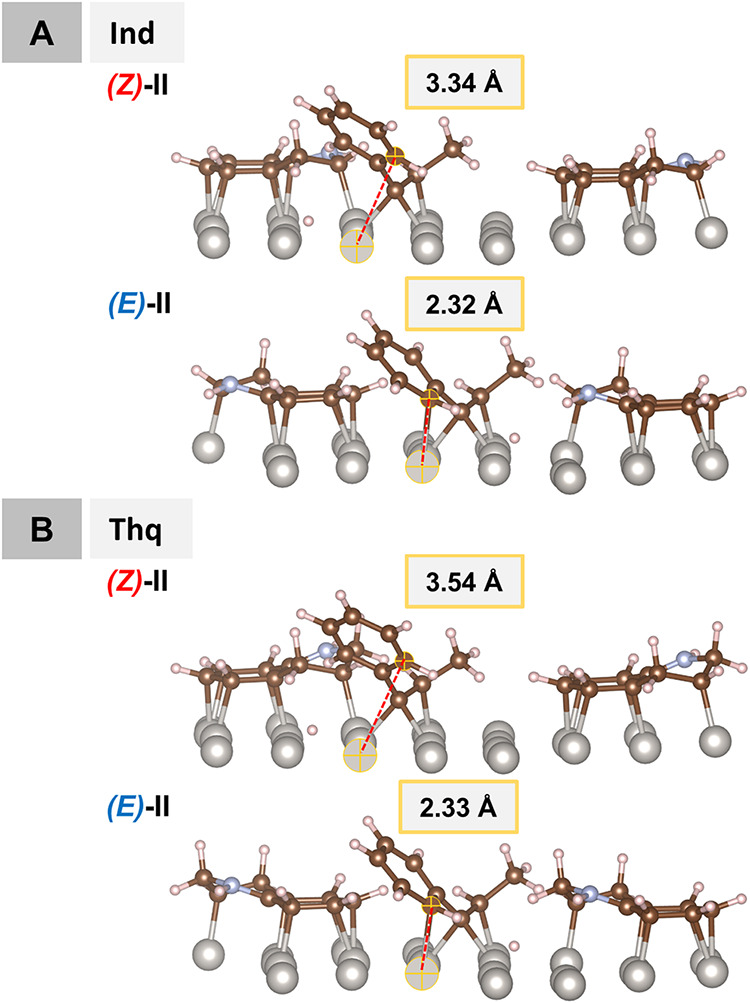
Visualization of the key bond distances of the lowest-energy conformer of *(Z)*- and *(E)*-forming intermediate state **II** of substrate **3** with Ind and Thq on Pt(111) surface.

## 3 Conclusions

This study provides a detailed mechanistic understanding of hydrogen-transfer reactions of internal alkynes using secondary amines as hydrogen donors on carbon-supported noble-metal catalysts. By integrating experimental results with theoretical modeling, we identified key factors governing both reactivity and selectivity, including the substrate electronic structure and adsorption behavior on catalytic surfaces.

High *(Z)*-selectivity toward the alkene intermediates, followed by sequential hydrogenation to fully saturated products, was consistently observed across the most active systems: Pt/C–Ind, Pd/C–Ind, Pt/C–Thq, and Pd/C–Thq. Kinetic analysis revealed strongly negative activation entropies, pointing to the formation of highly ordered transition states during the hydrogen transfer step.

Theoretical analysis confirmed that both electronic distribution and adsorption energy of the alkyne substrates critically influence the reaction profile. Substrate **2**, in particular, showed enhanced nucleophilic alkyne character and the most negative binding energy—both correlating with its exceptional experimental reactivity.

Additionally, a compensation effect observed in the Arrhenius plots for substrate **1** suggests an underlying mechanistic uniformity across different catalyst systems, linking the activation energy and entropy changes. This reinforces that a model hydrogen transfer proceeds via a concerted mechanism involving a well-organized surface-bound transition state shaped by both electronic and steric factors.

## Supplementary Material



## Notes

Experimental procedures and materials, computational methods, representative GC spectra, kinetic measurements, determination of activation energy (*E*_a_), determination of activation enthalpy (Δ*H* ^‡^), activation entropy (Δ*S* ^‡^), reaction order determination of substrates, as well as DFT coordinates, and theoretical methods (PDF)
