# Supplementary material for: Hydrogen Transfer to Internal Alkynes Using Secondary Amines on Carbon-Supported Noble Metals
Source: ACS Phys Chem Au. 2026 Jun 16;6(4):887–900. doi: 10.1021/acsphyschemau.6c00058 (PMC13397458; doi:10.1021/acsphyschemau.6c00058)
Supplement: Supplementary file 1 [file pg6c00058_si_001.pdf]

Supporting Information

Hydrogen Transfer to Internal Alkynes Using Secondary Amines on Carbon-Supported Noble Metals

Katharina Konieczny,[a,‡] Tianyin Qiu,[b,‡] Jan Paul Menzel,[b] Jacqueline Maslack,[a] Victor S. Batista[b] and Eszter Baráth*[a]

[a]Leibniz-Institut für Katalyse e.V. (LIKAT), Albert Einstein Str. 29a, Rostock, D-18059, Germany
[b]Yale University, Department of Chemistry, 225 Prospect St., P.O. Box 208107, New Haven, CT 06520, USA

‡These authors contributed equally.
*E-mail: eszter.barath@catalysis.de

Table of Contents

**Experimental procedures, materials**	**S3**
**Mode of calculations**	**S5**
**Representative GC spectra**	**S5**
**Kinetic measurements, determination of activation parameters**	**S6**
**Determination of activation enthalpy ($\Delta H^{‡}$) and activation entropy ($\Delta S^{‡}$)**	**S9**
**Reaction order determination of substrate 1**	**S9**
**Reaction order determination of substrate 2**	**S11**
**Reaction order determination of substrate 3**	**S12**
**H_2 evaluation**	**S12**
**Computational details**	**S14**
**References**	**S25**

Experimental procedures, materials

Chemicals The following chemicals were used as received, without any additional purification or further treatment: tetrahydroquinoline ((Thq), 98 %, *Sigma-Aldrich)*, indoline ((Ind), 99 %, *Sigma-Aldrich*), diisopropylethylamine (98 %, *Sigma-Aldrich*), diisopropylamine (95.5 %, *Sigma-Aldrich*), diphenylacetylene (98 %, *TCI*), 1-phenyl-1-propyne (98 %, *abcr*), methyl phenylpropiolate (97 %, *Sigma-Aldrich*) (98 %, *Thermo Scientific*), *trans*-stilbene (98 %, *TCI*), *cis*-stilbene (96.0 %, Sigma-Aldrich), 1,2-diphenylethane (96.0 %, *Sigma-Aldrich* 96.0 %), *cis/trans*-1-phenyl-1-propene (> 95 %, *TCI*), *trans*-1-phenyl-1-propene (99 %, Sigma-Aldrich), 1-phenylpropane (98 %, Sigma-Aldrich), methyl *cis/trans*-cinnamate (99 %, *Sigma-Aldrich*), methyl *trans*-cinnamate (99 %, *Sigma-Aldrich*), methyl 3-phenylpropionate (98 %, *Thermo Scientific*), anhydrous *p*-xylene (99 %, *Sigma-Aldrich*), mesitylene (99 %, *Sigma-Aldrich*), Pt/C (A and B sample) (10 wt% loading, *Sigma-Aldrich 205958*), Pd/C (10 wt% loading, *Sigma-Aldrich 205699*).

Metal catalysts activation The commercial metal catalysts (Pt/C, Pd/C) were activated before usage. The reduction was performed at 120 °C for 1 h hour, then the temperature was increased to 450 °C with a heating rate of 2 °C min^{-1} and held for 3 hours. The flow rate of H_2/N_2 (70/30) was 100 mL min^{-1}. The samples were cooled to room temperature under N_2-flow before collection. Afterwards the catalysts were immediately transferred into the glovebox with a brief exposure to air.

Catalytic reactions All reactions were performed with the same molar amount of reactant, catalyst and solvent under Argon atmosphere in a 10 mL Schlenk-tube. 0.05 mmol of metal catalyst (Pt/C (10 wt%): 97.5 mg or Pd/C (10wt%); 53.2 mg) was added to the Schlenk-tube in the glove box; after that 0.5 mmol of substrate (methylphenylpropiolate (**1**); diphenylacetylene (**2**); 1-phenyl-1-propyne (**3**)), 2.2 mmol of amine and 1.5 mL of anhydrous solvent were added outside the glove box under inert conditions. The reaction mixture was kept under inert conditions and heated to the corresponding temperature (reaction temperature 140°C; temperatures for kinetic investigations: 80 °C, 90 °C, 100 °C; 110 °C, 120 °C). After the required reaction time the reaction mixture was cooled down to room temperature (reaction time 5 h; different time regimes for kinetic measurements). 1 mL of the reaction mixture was removed and filtered. The aliquot (100 µL) was taken and diluted with 1 mL of *p*-xylene. The samples were analyzed by GC or GC-MS to gain the product distribution. (For the cross-checking of quantitative analysis of the product distribution mesitylene (2 µL) was used as internal standard.)

Note: Due to multiple experiments the usage of two Pt/C batches was necessary, please see characterization dataset in the manuscript part and in the upcoming sections of the supporting information.
Pt/C (A) was used for subsequent consolidated reactions, these are shown in: **Table S1**, **Table S3**; **Table S11**, **Table S13**, **Figure S4**, **Figure S6**.
Pt/C (B) was used for subsequent consolidated reactions, these are shown in: **Table S5**, **Table S6**, **Table S7**, **Table S8**, **Table S9**, **Table S15**, **Table S16**, **Figure S8**, **Figure S9**.

Recycling experiment The catalyst recycling test has been done using substrate **2** (0.5 mmol), diisopropylamine (2.2 mmol), Pt/C 10 wt% (batch B, 0.05 mmol metal) in *p*-xylene as solvent (1.5 mL) at 140 °C for 5 h reaction time according to the standard catalytical reaction protocol. After the first catalytic run and full precipitation of the catalyst the supernatant was removed with a syringe and checked with the above summarized GC protocol. All reactants (substrate, amine, solvent) were added to the catalyst, and the second catalytic run was carried out.

GC Quantification and qualification of the hydrogen-transfer reactions was analyzed by GC (*Agilent Technologies 7890 B GC*, column: *Agilent 19091J-413 HP-5* (30 m × 320 µm × 0.25 µm) equipped with a flame ionization detector (FID). The liquid sample (1 µL) was injected into the column. The temperature was initially held at 50 °C and increased with 4 °C min^{-1} to 70 °C, further heating with a rate of 1 °C min^{-1} from 70 °C to 80 °C was performed, followed by heating with a rate of 15 °C min^{-1} from 80 °C to 150 °C. The final heating section was from 150 °C to 300 °C with a rate of 25 °C min^{-1}. Identification of the components was performed by using the retention times of commercially available pure substances. Quantification of reactants was analyzed by the FID-signal. All Data was analyzed with *GC OpenLab Software*, *Version 1.9.0*, *Agilent Technologies (2001 - 2013)*.

GC-MS Quantification and qualification of the hydration reactions was analyzed by GC-MS (*Agilent Technologies 8860 GC*, column: *Agilent 19091J-413* (30 m × 320 µm × 0.25 µm) and MSD 5977) The liquid sample (1 µL) was injected into the column. The temperature was initially held at 50 °C and increased with 4 °C min^{-1} to 70 °C, further heating with a rate of 1 °C min^{-1} from 70 °C to 80 °C was performed, followed by heating with a rate of 15 °C min^{-1} from 80 °C to 150 °C. The final heating section was from 150 °C to 300 °C with a rate of 25 °C min^{-1}. Identification of the components was performed by using the retention times of commercially available pure substances. Quantification of reactants was analyzed by the FID-signal. Data was analyzed with *MassHunter Workstation Software*, *Agilent Mass Hunter Qualitative Analysis* 12.0.

ICP The metal content of the catalyst was measured by ICP-OES (*Varian/Agilent 715-ES*). Solutions of the samples are prepared, using the conventional techniques of quantitative and qualitative chemical analysis.

TEM measurements were performed on a *JOEL JEM-ARM200F* equipment. The average particle size and its standard deviation was calculated based on the Pt and Pd particle size distribution of at least 300 metal particles measured in at least five different particle domains of the catalyst sample.

H_2 Chemisorption The active carbon supported metal (Pt, Pd) was pre-treated at 573 K under 0.1 MPa H_2 for 1 h, followed by evacuation in vacuum for 1h. After the temperature was cooled to 298 K, the H_2 chemisorption and physisorption were subsequently determined in a pressure of hydrogen from 5 to 350 Torr. Then, the physisorbed H_2 was removed by outgasing the sample at 298 K for 1 h. The concentration of chemisorbed hydrogen on the metal was obtained by extrapolating the isotherm to zero Torr of H_2 pressure. The metal (Pt, Pd) dispersion and TOF were deduced by assuming an average H/metal ration of 1.

BET surface analysis Specific surface area of the support was determined from nitrogen adsorption-desorption isotherms recorded on a micromeritics ASAP 2020. The specific surface areas were calculated by applying the *B.E.T. theory.*

XRD powder pattern were recorded on a *Panalytical X'Pert* θ/2θ -diffractometer equipped with *Xcelerator* detector using automatic divergence slits and Cu kα1/α2 radiation (40 kV, 40 mA; λ= 0.15406 nm, 0.154443 nm). Cu beta-radiation was excluded using a nickel filter foil. Finely pestled samples were mounted on silicon zero background holders and data collection were performed with 0.086 °s^{-1} (25s), 0.021 °s^{-1} (100 s) or 0.005 °s^{-1} (400 s), respectively. Obtained intensities were converted from automatic to fixed divergence slits (0.25 °) for further analysis. Peak positions and profile were fitted with Pseudo-Voigt function using the HighScore Plus software package (*Panalytical*). Phase identification was done by using the PDF-2 database of the International Center of Diffraction Data (ICDD). (For XRD analysis of Pt/C, sample A was used.)

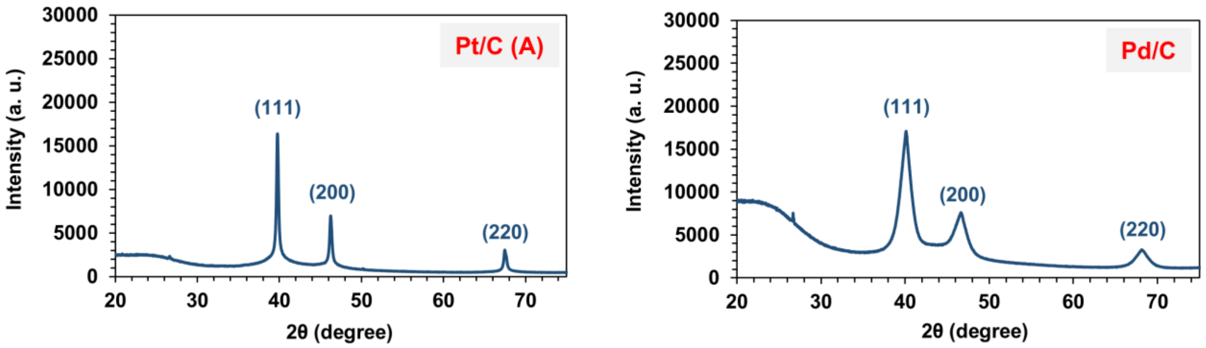

Figure S1. XRD pattern of Pt/C (A) and Pd/C.

Particle size distribution of Pt/C (A and B) and Pd/C

For the particle size distribution each metal was estimated based on at least 300 metal particles from at least 5 different particle domains of each catalyst. The average particle size is 5.0 ± 2.0 nm for Pt/C (A), 3.6 ± 2.7 nm for Pt/C (B) and 5.7 ± 1.1 nm for Pd/C, respectively.

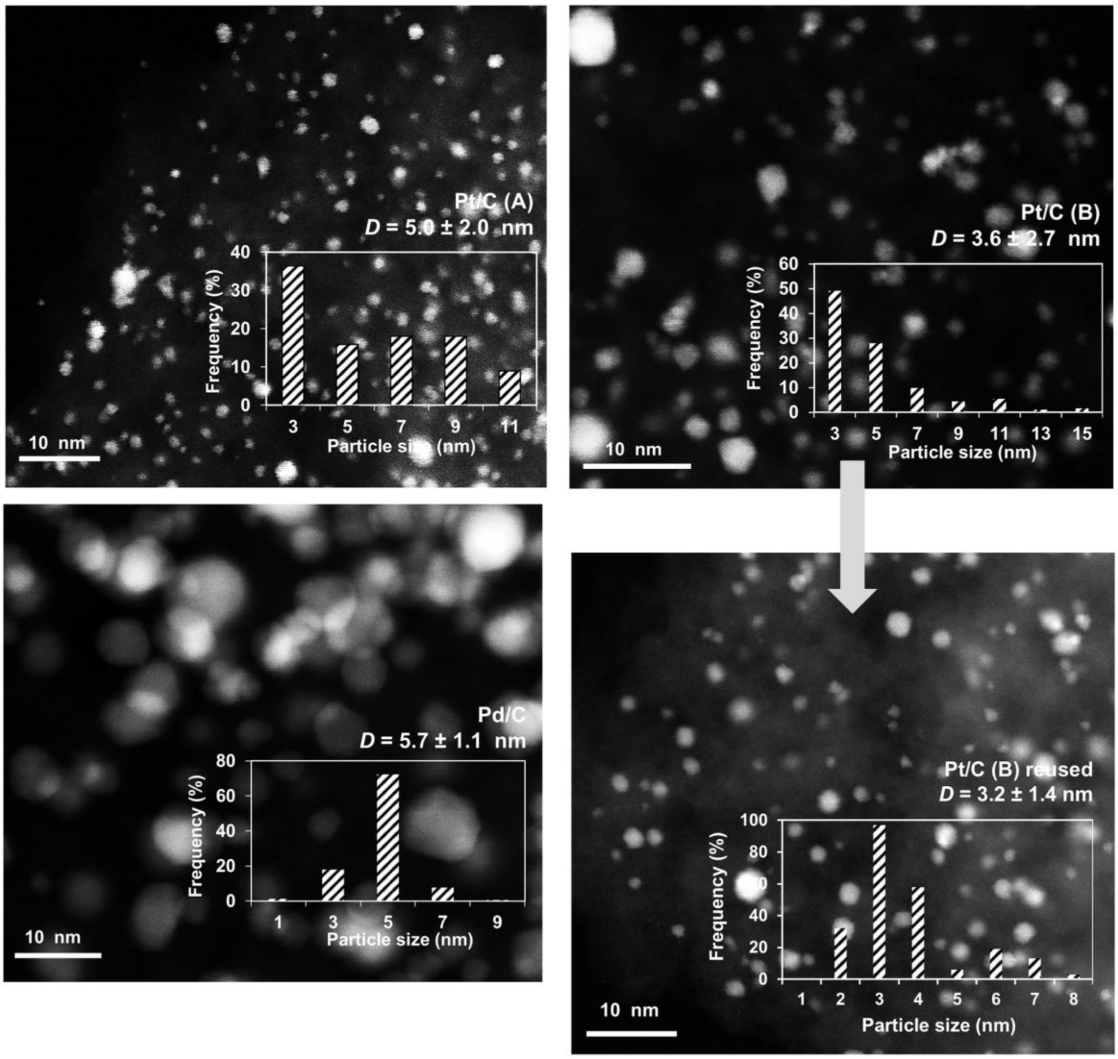

Figure S2. Particle size distribution of Pt/C (A and B), Pd/C and the reused Pt/C (B) catalyst *via* TEM analysis.

Mode of calculations

Conversion = (mole of converted reactant / mole of the starting reactant) (× 100 (%)); **Yield** = the ration of the amount of reaction product and the amount of the starting material (× 100 (%)); **Selectivity** = the ratio of the amount of reaction product and the amount of a converted feedstock material (× 100 (%)); **Initial reaction rate** was deduced from the slope of the linear fit to the conversion versus reaction time plot in the linear region at low conversion; **TOF** = mole of converted reactant / (mole of accessible metal sites × reaction time) (mol $mol_{(surf. metal)}^{-1}$ s^{-1} which is shortened as s^{-1}); **Accessible metal sites** (mol $g_{(cat)}^{-1}$ were calculated by the normalization of the catalyst amount to metal dispersion and metal loading.

Representative GC spectra

Representative GC spectra (without purification) of substrates **1-3** are given in **Figure S3** including their corresponding saturated derivatives. Methyl phenylpropiolate (**1**) (**Figure S3 A**), diphenylacetylene (**2**) (**Figure S3 B**), 1-phenyl-1-propyne (**3**) (**Figure S3 C**). GC spectra of amine donors are given including their corresponding oxidized derivative. Indoline (**Figure S3 A**), tetrahydroquinoline (**Figure S3 D**).

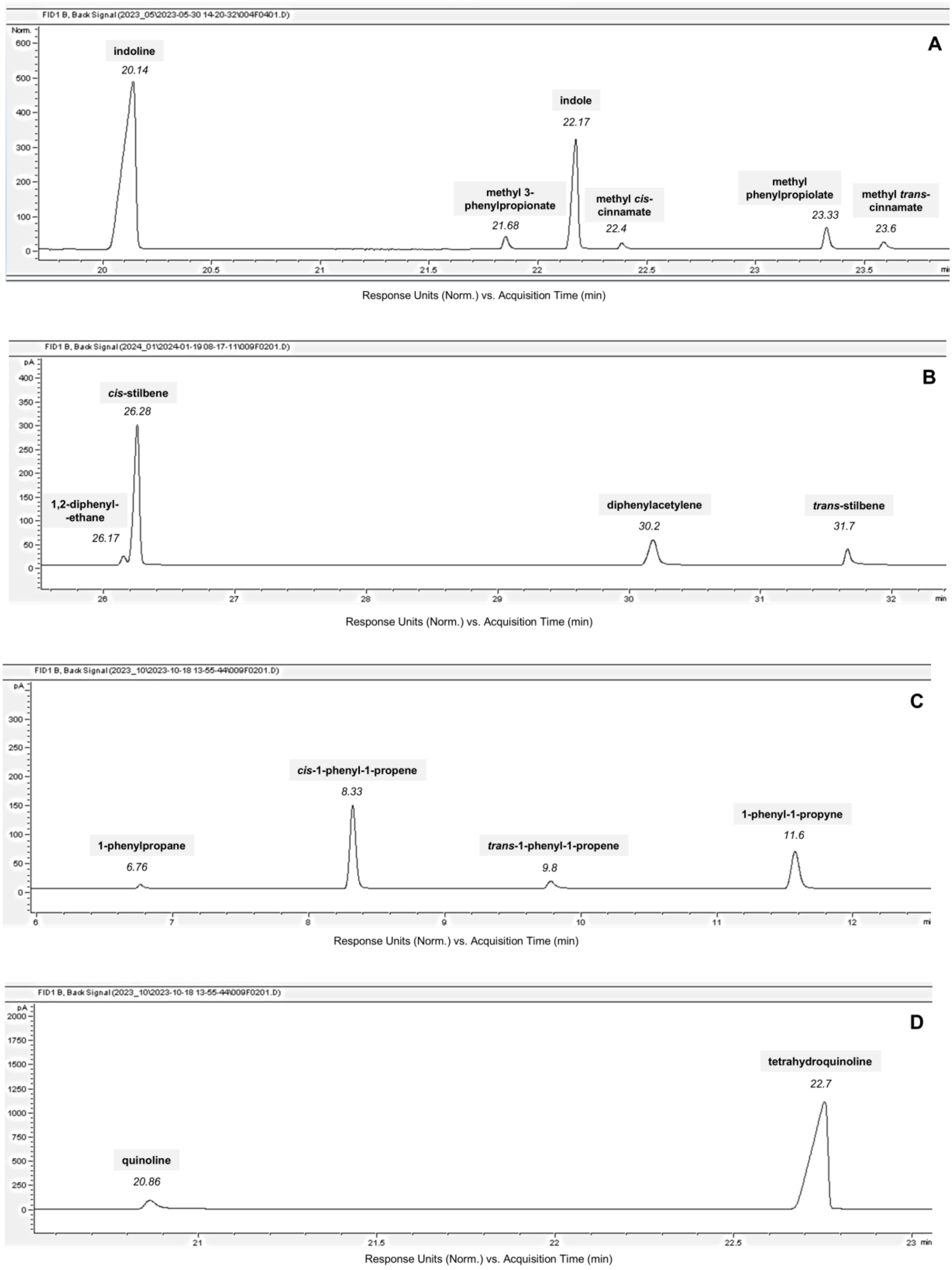

Figure S3. Representative GC spectra of substrate **1-3** and their corresponding alkene and alkane derivatives.

Kinetic measurements, determination of activation parameters

Hydrogen transfer of substrate **1-3** over Pt/C and Pd/C with indoline or Thq. Reaction conditions: substrate (0.5 mmol), *p*-xylene (1.5 mL), amine (2.2 mmol) over Pt/C (10 wt%, 0.05 mmol Pt) or Pd/C (10 wt%, 0.05 mmol Pd), under Ar at a given reaction temperature and reaction time. All data points of kinetic experiments were taken from separate measurements, no *in situ* sampling was applied.

Table S1. Initial rates at different reaction temperatures for substrate **1** with indoline catalyzed by Pt/C.

T (°C)	T (K)	T^{-1} (K^{-1})	r (mol $g_{(cat)}^{-1}$ s^{-1})	ln(r)	TOF (mol $mol_{(surf.\ metal)}^{-1}$ s^{-1})	ln(TOF)
140	413	2.42×10^{-3}	2.44×10^{-6}	-12.92	2.28×10^{-2}	-3.78
120	393	2.54×10^{-3}	1.23×10^{-6}	-13.61	1.16×10^{-2}	-4.46
100	373	2.68×10^{-3}	5.68×10^{-7}	-14.38	5.31×10^{-2}	-5.24
90	363	2.75×10^{-3}	2.65×10^{-7}	-15.14	2.48×10^{-3}	-6.00

Table S2. Initial rates at different reaction temperatures for substrate **1** with indoline catalyzed by Pd/C.

T (°C)	T (K)	T^{-1} (K^{-1})	r (mol $g_{(cat)}^{-1}s^{-1}$)	ln(r)	TOF (mol $mol_{(surf.\ metal)}^{-1}s^{-1}$)	ln(TOF)	ln(A)
140	413	2.42×10^{-3}	3.85×10^{-7}	-14.77	2.48×10^{-3}	-6.00	9.16
120	393	2.54×10^{-3}	1.84×10^{-7}	-15.51	1.18×10^{-3}	-6.74	
110	383	2.61×10^{-3}	1.45×10^{-7}	-15.75	9.33×10^{-4}	-6.98	
100	373	2.68×10^{-3}	7.05×10^{-8}	-16.47	4.54×10^{-4}	-7.70	

Table S3. Initial rates at different reaction temperatures for substrate **1** with Thq catalyzed by Pt/C.

T (°C)	T (K)	T^{-1} (K^{-1})	r (mol $g_{(cat)}^{-1}s^{-1}$)	ln(r)	TOF (mol $mol_{(surf.\ metal)}^{-1}$ s^{-1})	ln(TOF)	ln(A)
140	413	2.42×10^{-3}	2.15×10^{-7}	-15.35	2.01×10^{-3}	-6.21	5.58
120	393	2.54×10^{-3}	1.09×10^{-7}	-16.03	1.02×10^{-3}	-6.89	
110	383	2.61×10^{-3}	7.63×10^{-8}	-16.39	7.15×10^{-4}	-7.24	
100	373	2.68×10^{-3}	6.22×10^{-8}	-16.59	5.83×10^{-4}	-7.45	

Table S4. Initial rates at different reaction temperatures for substrate **1** with Thq catalyzed by Pd/C.

T (°C)	T (K)	T^{-1} (K^{-1})	r (mol $g_{(cat)}^{-1}s^{-1}$)	ln(r)	TOF (mol $mol_{(surf.\ metal)}^{-1}$ s^{-1})	ln(TOF)	ln(A)
140	413	2.42×10^{-3}	2.81×10^{-7}	-15.08	1.81×10^{-3}	-6.31	15.20
120	393	2.54×10^{-3}	1.19×10^{-7}	-15.94	7.67×10^{-4}	-7.17	
100	373	2.68×10^{-3}	2.85×10^{-8}	-17.37	1.84×10^{-4}	-8.60	

Table S5. Initial rates at different reaction temperatures for substrate **1** with (^{i}Pr)$_2$NEt catalyzed by Pt/C.

T (°C)	T (K)	T^{-1} (K^{-1})	r (mol $g_{(cat)}^{-1}s^{-1}$)	ln(r)	TOF (mol $mol_{(surf.\ metal)}^{-1}$ s^{-1})	ln(TOF)
140	413	2.42×10^{-3}	7.69×10^{-7}	-14.08	4.86×10^{-3}	-5.33
120	393	2.54×10^{-3}	2.59×10^{-7}	-15.17	1.63×10^{-3}	-6.42
100	373	2.68×10^{-3}	6.82×10^{-8}	-16.50	4.31×10^{-3}	-7.75

Table S6. Initial rates at different reaction temperatures for substrate **2** with indoline catalyzed by Pt/C.

T (°C)	T (K)	T^{-1} (K^{-1})	r (mol $g_{(cat)}^{-1}s^{-1}$)	ln(r)	TOF (mol $mol_{(surf.\ metal)}^{-1}$ s^{-1})	ln(TOF)
110	383	2.61×10^{-3}	5.75×10^{-6}	-12.07	3.63×10^{-2}	-3.32
100	373	2.68×10^{-3}	4.07×10^{-6}	-12.41	2.57×10^{-2}	-3.66
90	363	2.75×10^{-3}	2.35×10^{-6}	-12.96	1.48×10^{-2}	-4.21
80	353	2.83×10^{-3}	1.80×10^{-6}	-13.23	1.13×10^{-2}	-4.48

Table S7. Initial rates at different reaction temperatures for substrate **2** with Thq catalyzed by Pt/C.

T (°C)	T (K)	T^{-1} (K^{-1})	r (mol $g_{(cat)}^{-1}s^{-1}$)	ln(r)	TOF (mol $mol_{(surf.\ metal)}^{-1}$ s^{-1})	ln(TOF)
140	413	2.42×10^{-3}	2.36×10^{-6}	-12.96	1.49×10^{-2}	-4.20
120	393	2.54×10^{-3}	1.21×10^{-6}	-13.63	7.66×10^{-3}	-4.87
110	383	2.61×10^{-3}	7.34×10^{-7}	-14.12	4.65×10^{-3}	-5.37
100	373	2.68×10^{-3}	5.28×10^{-7}	-14.45	3.35×10^{-3}	-5.70

Table S8. Initial rates at different reaction temperatures for substrate **3** with indoline catalyzed by Pt/C.

T (°C)	T (K)	T^{-1} (K^{-1})	r (mol $g_{(cat)}^{-1}s^{-1}$)	ln(r)	TOF (mol $mol_{(surf.\ metal)}^{-1}$ s^{-1})	ln(TOF)
140	413	2.42×10^{-3}	5.57×10^{-6}	-12.10	3.52×10^{-2}	-3.35
120	393	2.54×10^{-3}	2.94×10^{-6}	-12.74	1.85×10^{-2}	-3.99
110	383	2.61×10^{-3}	2.21×10^{-6}	-13.02	1.40×10^{-2}	-4.27
100	373	2.68×10^{-3}	1.76×10^{-6}	-13.25	1.11×10^{-2}	-4.50
90	363	2.75×10^{-3}	1.35×10^{-6}	-13.51	8.55×10^{-3}	-4.76

Table S9. Initial rates at different reaction temperatures for substrate **3** with Thq catalyzed by Pt/C.

T (°C)	T (K)	T^{-1} (K^{-1})	r (mol $g_{(cat)}^{-1}s^{-1}$)	ln(r)	TOF (mol $mol_{(surf.\ metal)}^{-1}$ s^{-1})	ln(TOF)
140	413	2.42×10^{-3}	1.09×10^{-6}	-13.73	6.92×10^{-3}	-4.97
120	393	2.54×10^{-3}	5.52×10^{-7}	-14.41	3.49×10^{-3}	-5.66
110	383	2.61×10^{-3}	3.12×10^{-7}	-14.98	1.98×10^{-3}	-6.23
100	373	2.68×10^{-3}	2.22×10^{-7}	-15.32	1.41×10^{-3}	-6.57

Determination of activation enthalpy ($\Delta H^\ddagger$) and activation entropy ($\Delta S^\ddagger$)

The Eyring equation with the corresponding measured TOF values (in here k = TOF, if reaction order is 0) were used to calculate the activation entropy and enthalpy values (k_B = Boltzmann constant (1.38×10^{-23} J K^{-1}), T = temperature (K), h = Planck constant (6.63×10^{-34} J s), R = universal gas constant (8.314 J mol^{-1} K^{-1})).[1-3]

$$k = \frac{T \times k_B}{h} e^{-\frac{\Delta H^\ddagger - T\Delta S^\ddagger}{RT}}$$

Scheme S1. Linearization of the Eyring equation to determine activation enthalpy and activation entropy.

Table S10. Calculation of [ln (TOF h k_B^{-1} T^{-1}) R] values on Pt/C and Pd/C for the determination of activation entropy and activation enthalpy.

**Substrate**	**T (°C)**	**1/T (K^{-1})**	**Catalyst**				
			**Pt/C**			**Pd/C**	
			**Ind**	**Thq**	**(^{i}Pr)$_2$NEt**	**Ind**	**Thq**
			**ln (TOF h k_B^{-1} T^{-1}) R**				
**1**	140	2.42×10^{-3}	-279.06	-299.25	-291.92	-297.50	-300.11
	120	2.54×10^{-3}	-284.30	-304.50	-300.56	-303.23	-306.85
	110	2.61×10^{-3}	-	-307.22	-	-305.01	-
	100	2.68×10^{-3}	-290.32	-308.70	-311.21	-310.77	-318.30
	90	2.75×10^{-3}	-296.44		-	-	-
**2**	140	2.42×10^{-3}	-	-282.57	-	-	-
	120	2.54×10^{-3}	-	-287.72	-	-	-
	110	2.61×10^{-3}	-274.56	-291.65	-	-	-
	100	2.68×10^{-3}	-277.22	-294.17	-	-	-
	90	2.75×10^{-3}	-281.57	-	-	-	-
	80	2.83×10^{-3}	-283.57	-	-	-	-
**3**	140	2.42×10^{-3}	-275.45	-288.97	-	-	-
	120	2.54×10^{-3}	-280.36	-294.24	-	-	-
	110	2.61×10^{-3}	-282.51	-298.77	-	-	-
	100	2.68×10^{-3}	-284.20	-301.38	-	-	-
	90	2.75×10^{-3}	-286.14	-	-	-	-

Reaction order determination of substrate 1

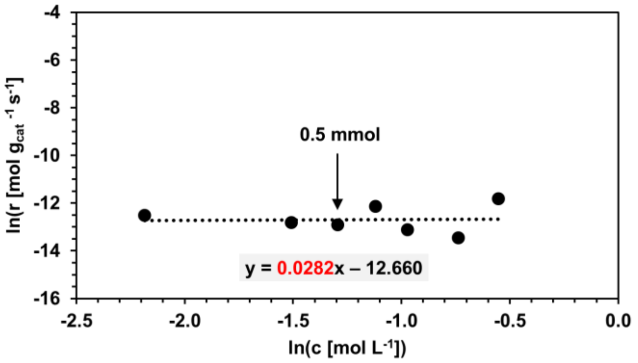

Figure S4. Determination reaction order of substrate **1** on Pt/C in the presence of indoline at 140 °C under inert conditions.

Table S11. Reaction order of methyl-3-phenylpropiolate (**1**) (substrate **1** (0.2-1.1 mmol), Pt/C (10 wt%, 0.05 mmol Pt), indoline (2.2 mmol), *p*-xylene (1.5 mL), 140 °C, under Ar and atmospheric pressure).

n (mol)	c (mol L^{-1})	ln(c)	r (mol g_{cat}^{-1} s^{-1})	ln(r)
0.0002	0.113	-2.184	3.62×10^{-6}	-12.53
0.0004	0.222	-1.507	2.69×10^{-6}	-12.83
0.0005	0.275	-1.292	2.44×10^{-6}	-12.92
0.0006	0.327	-1.118	5.29×10^{-6}	-12.15
0.0007	0.378	-0.972	1.99×10^{-6}	-13.13
0.0009	0.479	-0.736	1.41×10^{-6}	-13.47
0.0011	0.576	-0.551	7.33×10^{-6}	-11.82

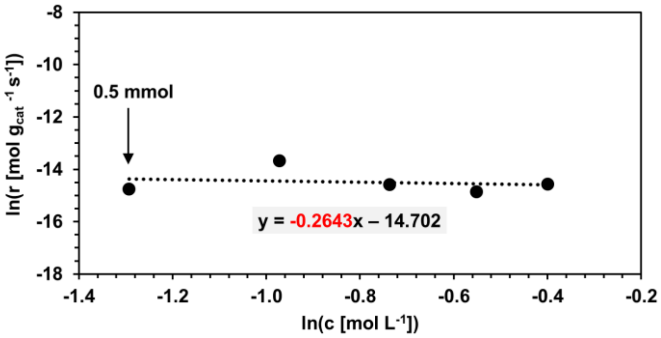

Figure S5. Determination reaction order of substrate **1** on Pd/C in the presence of indoline at 140 °C under inert conditions.

Table S12. Reaction order of methyl-3-phenylpropiolate (**1**) (substrate **1** (0.5-1.3 mmol), Pd/C (10 wt%, 0.05 mmol Pd), indoline (2.2 mmol), *p*-xylene (1.5 mL), 140 °C, under Ar and atmospheric pressure).

n (mol)	c (mol L^{-1})	ln(c)	r (mol g_{cat}^{-1} s^{-1})	ln(r)
0.0005	0.275	-1.292	3.85×10^{-7}	-14.77
0.0007	0.378	-0.972	1.15×10^{-6}	-13.68
0.0009	0.479	-0.736	4.64×10^{-7}	-14.58
0.0011	0.576	-0.551	3.52×10^{-7}	-14.86
0.0013	0.671	-0.399	4.70×10^{-7}	-14.57

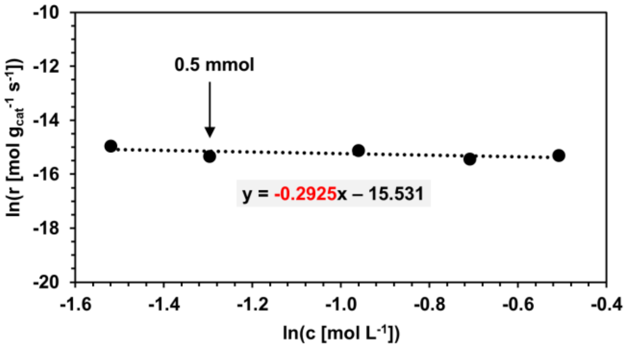

Figure S6. Determination reaction order of substrate **1** on Pt/C in the presence of Thq at 140 °C under inert conditions.

Table S13. Reaction order of methyl-3-phenylpropiolate (**1**) (substrate **1** (0.4-1.1 mmol), Pt/C (10 wt%, 0.05 mmol Pt), Thq (2.2 mmol), *p*-xylene (1.5 mL), 140 °C, under Ar and atmospheric pressure).

n (mol)	c (mol L^{-1})	ln(c)	r (mol g_{cat}^{-1} s^{-1})	ln(r)
0.0004	0.219	-1.519	3.18×10^{-7}	-14.96
0.0005	0.274	-1.296	2.15×10^{-7}	-15.35
0.0007	0.383	-0.960	2.70×10^{-7}	-15.12
0.0009	0.493	-0.708	1.97×10^{-7}	-15.44
0.0011	0.602	-0.508	2.23×10^{-7}	-15.31

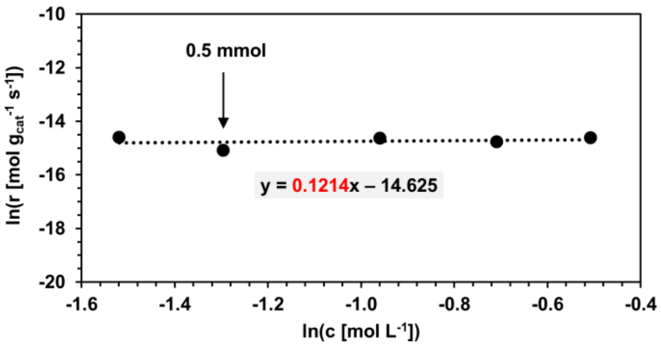

Figure S7. Determination reaction order of substrate **1** on Pd/C in the presence of Thq at 140 °C under inert conditions.

Table S14. Reaction order of methyl-3-phenylpropiolate (**1**) (substrate **1** (0.4-1.1 mmol), Pd/C (10 wt%, 0.05 mmol Pd), Thq (2.2 mmol), *p*-xylene (1.5 mL), 140 °C, under Ar and atmospheric pressure).

n (mol)	c (mol L^{-1})	ln(c)	r (mol g_{cat}^{-1} s^{-1})	ln(r)
0.0004	0.219	-1.519	4.54×10^{-7}	-14.60
0.0005	0.274	-1.296	2.81×10^{-7}	-15.08
0.0007	0.383	-0.960	4.40×10^{-7}	-14.64
0.0009	0.493	-0.708	3.82×10^{-7}	-14.78
0.0011	0.602	-0.508	4.45×10^{-7}	-14.63

Reaction order determination of substrate 2

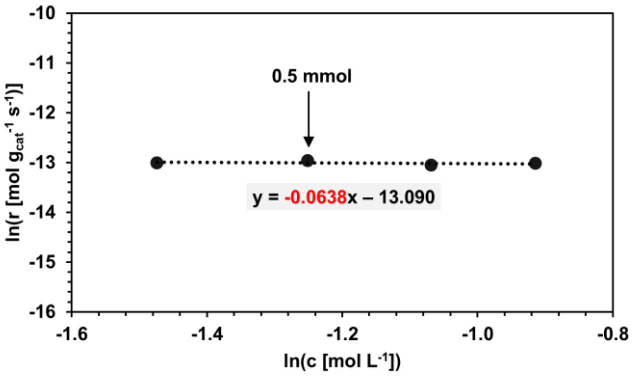

Figure S8. Determination reaction order of substrate **2** on Pt/C in the presence of indoline at 90 °C under inert conditions.

Table S15. Reaction order of diphenylacetylene (**2**) (substrate **2** (0.4-0.7 mmol), Pt/C (10 wt%, 0.05 mmol Pt), indoline (2.2 mmol), *p*-xylene (1.5 mL), 90 °C, under Ar and atmospheric pressure).

n (mol)	c (mol L^{-1})	ln(c)	r (mol g_{cat}^{-1} s^{-1})	ln(r)
0.0004	0.229	-1.474	2.23×10^{-6}	-13.01
0.0005	0.286	-1.251	2.35×10^{-6}	-12.96
0.0006	0.344	-1.068	2.13×10^{-6}	-13.06
0.0007	0.401	-0.914	2.21×10^{-6}	-13.02

Reaction order determination of substrate 3

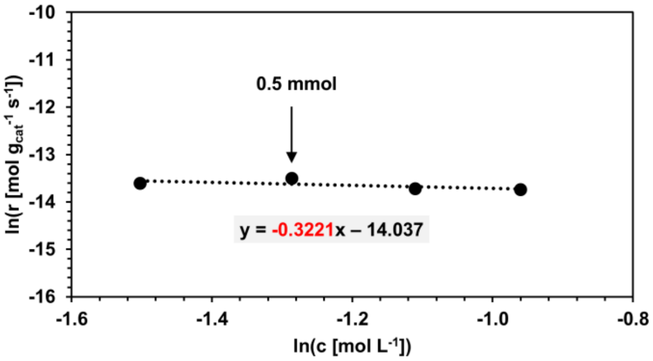

Figure S9. Determination reaction order of substrate **3** on Pt/C in the presence of indoline at 90 °C under inert conditions.

Table S16. Reaction order of 1-phenyl-1-propyne (**3**) (substrate 3 (0.4-0.7 mmol), Pt/C (10 wt%, 0.05 mmol Pt), indoline (2.2 mmol), *p*-xylene (1.5 mL), 90 °C, under Ar and atmospheric pressure).

n (mol)	c (mol L^{-1})	ln(c)	r (mol g_{cat}^{-1} s^{-1})	ln(r)
0.0004	0.223	-1.502	1.23×10^{-6}	-13.61
0.0005	0.276	-1.286	1.35×10^{-6}	-13.51
0.0006	0.330	-1.110	1.10×10^{-6}	-13.72
0.0007	0.383	-0.960	9.40×10^{-7}	-13.88

H_2 evaluation

Table S17. Olefin and oxidized amine formation using Pt/C (corresponding oxidized amines are 1*H*-indole and quinoline).

Entry	Substrate (0.5 mmol)	Catalyst	Amine (2.2 mmol)	Reaction temperature (°C)	Initial rate (mol $g_{(cat)}^{-1}$ s^{-1})	
					Oxidized amine formation (calculated from the yield (%))	Olefin formation (calculated from the conversion (%))[a]
1	1	Pt/C	Ind	140	2.59×10^{-6}	2.44×10^{-6}
2	1		Thq	140	1.37×10^{-7}	2.15×10^{-7}
3	2		Ind	110	3.86×10^{-6}	5.75×10^{-6}
4	2		Thq	140	3.26×10^{-7}	2.36×10^{-6}
5	3		Ind	140	1.97×10^{-6}	5.57×10^{-6}
6	3		Thq	140	8.05×10^{-8}	1.09×10^{-6}

[a] The corresponding rate values of olefin formation are from **Table S1**, **S3**, **S6-S9**.

Note: In order to analyze the H_2 formation and its presence in the gas phase, we cross-checked the transfer hydrogenation of methylphenylpropiolate (0.5 mmol) at 140 °C, using Pt/C (10 wt%, 0.05 mmol, batch B) and indoline as H-source (2.2 mmol) in *p*-xylene (1.5 mL), at different reaction times (**Table S18**, entry 1-2). We also carried out the same measurement **without** the substrate (**Table S18**, entry 3). For completeness, we also summarized the GC characteristics in **Table S19**.

Table S18. GC analysis of the gas/liquid phase H_2 evaluation.

**Amine conversion to 1*H*-indole (%)**	**Theoretical H_2 amount from amine (mmol)**	**Converted substrate to alkene (2H consumed) (mmol)**	**Converted substrate to alkane (4H consumed) (mmol)**	**Incorporated 2H in the substrate (mmol)**	**Excess H_2 in sample (mmol)**	**Unreacted H_2 in gas phase (%)**	**Calculated unreacted non-gaseous hydrogen (%)**
26.3 (after 5 min)	0.5786	0.3445	0.02	0.3845	0.1941	0.0	100
81.3 (after 1 h)	1.7886	0	0.5	1	0.7886	2.1	98
13.9 (after 5 min)	0.3058	0	0	0	0.3058	1.6	98

Measurement conditions: analysis of the composition of the headspace was performed using a GC (Agilent 6890) with equipped CP-Molecular sieves 5 Å porous layer open tubular (PLOT) column. Calibration was done using certified gas mixtures (Air Liquide) as standards. The progress of the catalytical performance was analyzed by GC: Agilent 19091J-413 HP-5 equipped with a flame ionization detector.

Table S19. GC characteristics of the gas phase measurement.

**Entry**	**Amine conversion to 1*H*-indole (%)**	**Reaction time (min)**	**H_2 amount analyzed by GC (mmol)**	**H_2 amount extrapolated in headspace (mmol)**
**1**	26.3	5	2.0128×10^{-9}	6.8436×10^{-5}
**2**	81.3	60	4.8730×10^{-7}	0.01657
**3**	13.9	5	1.4178×10^{-7}	0.00482

Measurement conditions: analysis of the composition of the headspace was performed using a GC (Agilent 6890) with equipped CP-Molecular sieves 5 Å porous layer open tubular (PLOT) column. Calibration was done using certified gas mixtures (Air Liquide) as standards. The progress of the catalytical performance was analyzed by GC: Agilent 19091J-413 HP-5 equipped with a flame ionization detector.

Computational details

Density functional theory (DFT) calculations were performed using the Vienna Ab initio Simulation Package (VASP) with the projector augmented wave (PAW) method.[4-8] The Perdew-Burke-Ernzerhof (PBE) exchange-correlation functional with D3-BJ dispersion corrections was employed.[9,10] A plane-wave energy cutoff of 500 eV was used, and Brillouin-zone sampling was conducted on a Γ-centered 3×3×1 Monkhorst-Pack k-point grid.[11] Self-consistent calculations were converged to an energy threshold of 10^{-6} eV. For the gas phase molecules, the same settings were applied, using a simulation box size of 25x25x25 Å^3 and a 1x1x1 k-point grid. The slab models were constructed as follows: First, bulk geometry optimization was performed on a Γ-centered 11×11×1 Monkhorst-Pack k-point grid, allowing the periodic cell dimensions to relax freely. From the optimized bulk structure, (111) surfaces were created, consisting of four atomic layers with a 6×6 arrangement in each layer. The bottom two layers were constrained to the bulk geometry, while the upper layers were allowed to relax to capture surface relaxation effects. The resulting simulation box is shown in **Figure S10**. Visualizations were generated using VESTA.[12]

Additionally, Gaussian 09 calculations were conducted on the gas phase alkynes using the B3LYP functional with the 6-31G(d,p) basis set and Grimme's D3 dispersion corrections (GD3)[13-16]. Electrostatic potential distributions were evaluated in the presence of a polarizable continuum model (PCM) representing water as the solvent.[17] Mulliken charges were calculated to assess partial charges,[18] particularly focusing on alkyne carbon atoms. Gaussian visualizations were created using GaussView.[19]

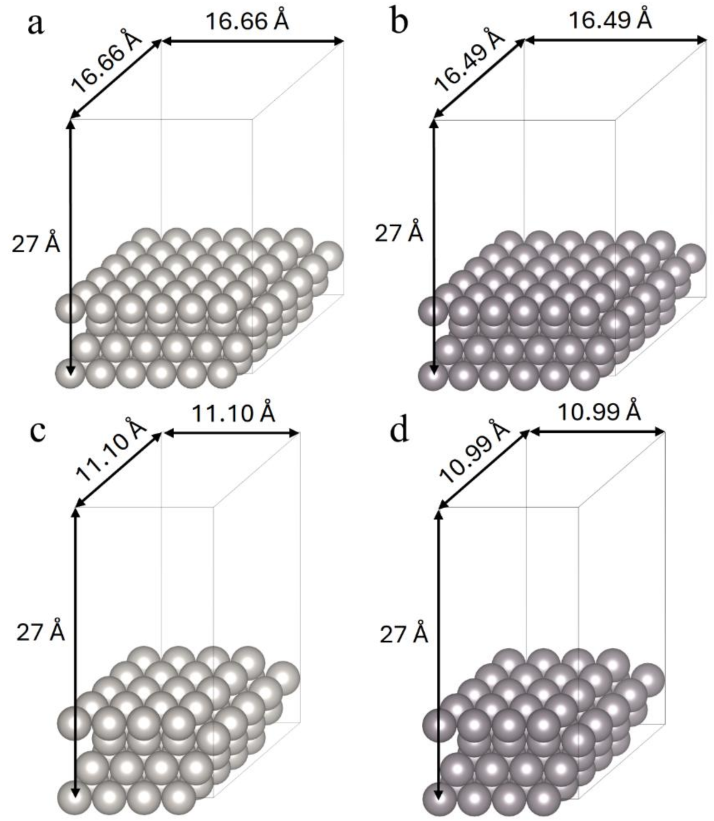

Figure S10. Simulation boxes of Pd(111) and Pt(111) surfaces used in this study. Panels (**a**) and (**b**) show the larger (6 × 6 × 4 layer) slabs of Pt and Pd, respectively, used for calculating adsorption binding energies under low coverage conditions. Panels (**c**) and (**d**) display the smaller (4 × 5 × 4 layer) slabs for Pt and Pd, respectively, which were employed in mechanistic studies under high surface coverage. All slabs are cleaved along the (111) orientation, and the box dimensions are labeled accordingly.

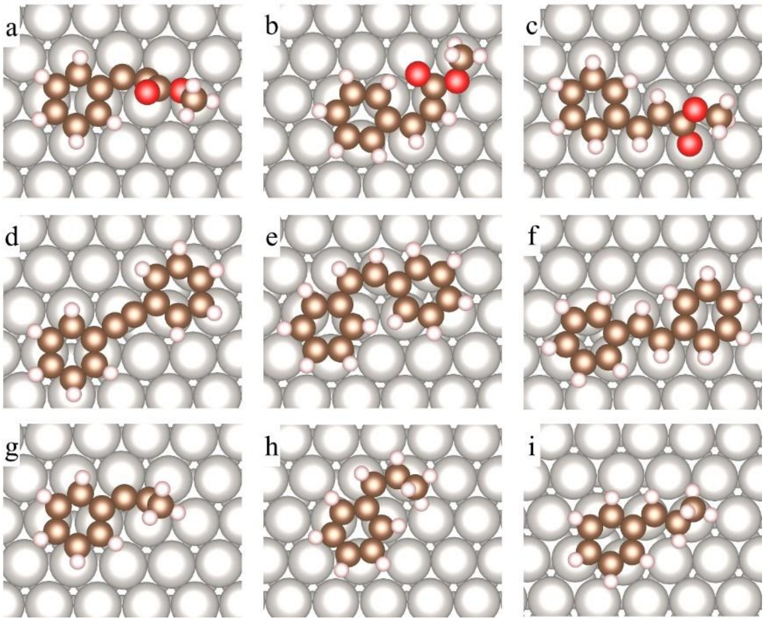

Figure S11. Lowest-energy binding conformations of the alkyne and alkene intermediates on the Pt(111) surface for three different substrates. Panels (**a–c**) correspond to substrate **1**, (**d–f**) to substrate **2**, and (**g–i**) to substrate **3**. Within each set, the structures represent the adsorbed states of the alkyne (**a**, **d**, **g**), *cis*-alkene (**b**, **e**, **h**), and *trans*-alkene (**c**, **f**, **i**) intermediates. All conformations are optimized to their respective minima on the Pt(111) slab.

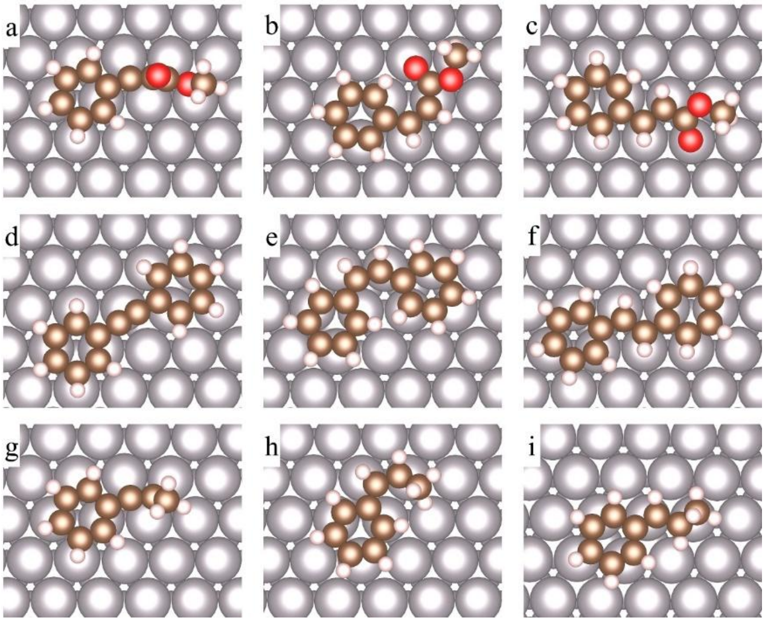

Figure S12. Lowest-energy binding conformations of the alkyne and alkene intermediates on the Pd(111) surface for three different substrates. Panels (**a–c**) correspond to substrate **1**, (**d–f**) to substrate **2**, and (**g–i**) to substrate **3**. Within each set, the structures represent the adsorbed states of the alkyne (**a**, **d**, **g**), *cis*-alkene (**b**, **e**, **h**), and trans-alkene (**c**, **f**, **i**) intermediates. All conformations are optimized to their respective minima on the Pd(111) slab.

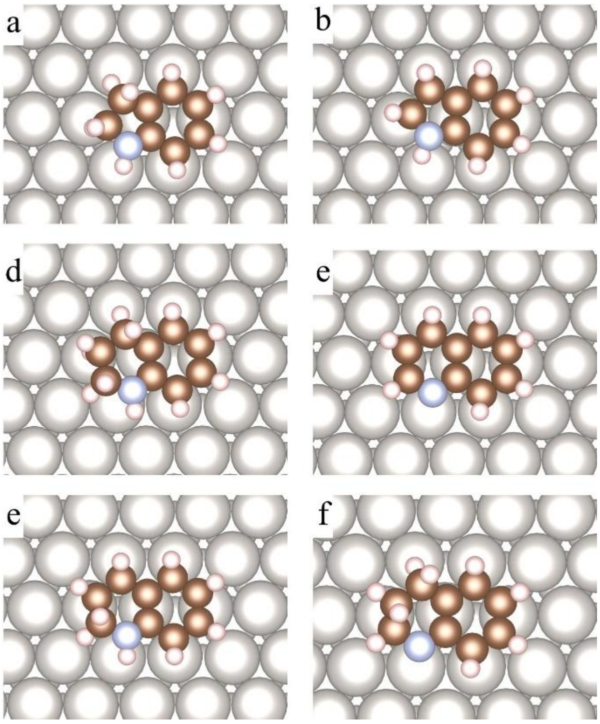

Figure S13. Top-view binding conformations of various amines adsorbed on the Pt(111) surface. Panels (**a–f**) correspond to the lowest-energy geometries for indoline (Ind), 1H-Indole (HInd), tetrahydroquinoline (Thq), quinoline (Quin), 1,2-dihydroquinoline (1,2-Di), and 3,4-dihydroquinoline (3,4-Di), respectively. Hydrogen, carbon, nitrogen, and platinum atoms are shown in white, brown, blue, and silver, respectively. These structures represent key intermediates relevant to the reaction mechanism under surface-bound conditions.

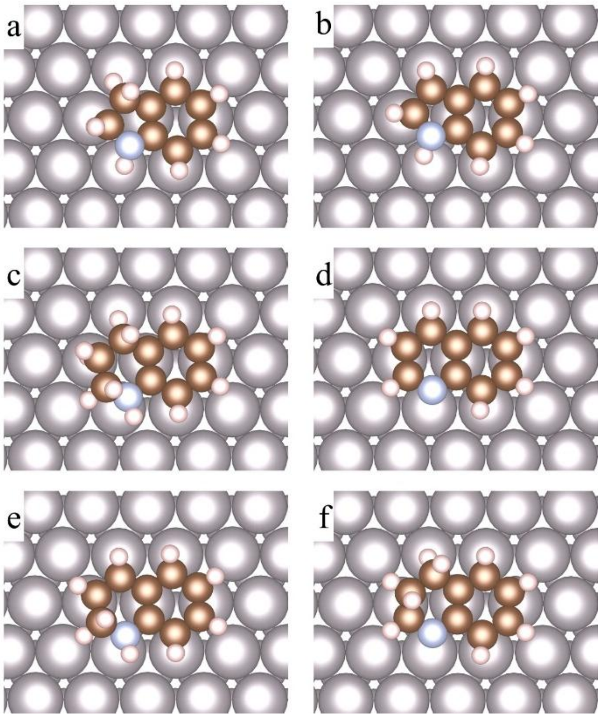

Figure S14. Top-view binding conformations of various amines adsorbed on the Pd(111) surface. Panels (**a–f**) correspond to the lowest-energy geometries for indoline (Ind), 1H-Indole (HInd), tetrahydroquinoline (Thq), quinoline (Quin), 1,2-dihydroquinoline (1,2-Di), and 3,4-dihydroquinoline (3,4-Di), respectively. Hydrogen, carbon, nitrogen, and palladium atoms are shown in white, brown, blue, and gray, respectively. These structures represent key intermediates relevant to the reaction mechanism under surface-bound conditions.

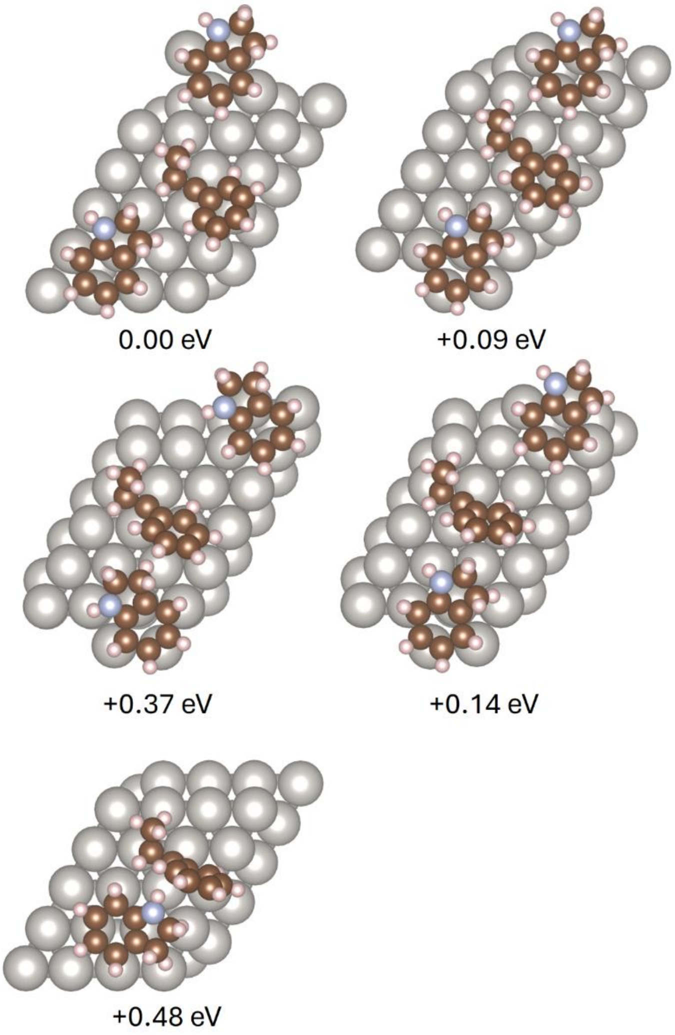

Figure S15. Alternative conformations for the starting state (**I**) of the *cis*-forming reaction complex with Ind (indoline) on Pt(111) surface. Energies are normalized on the lowest energy conformer.

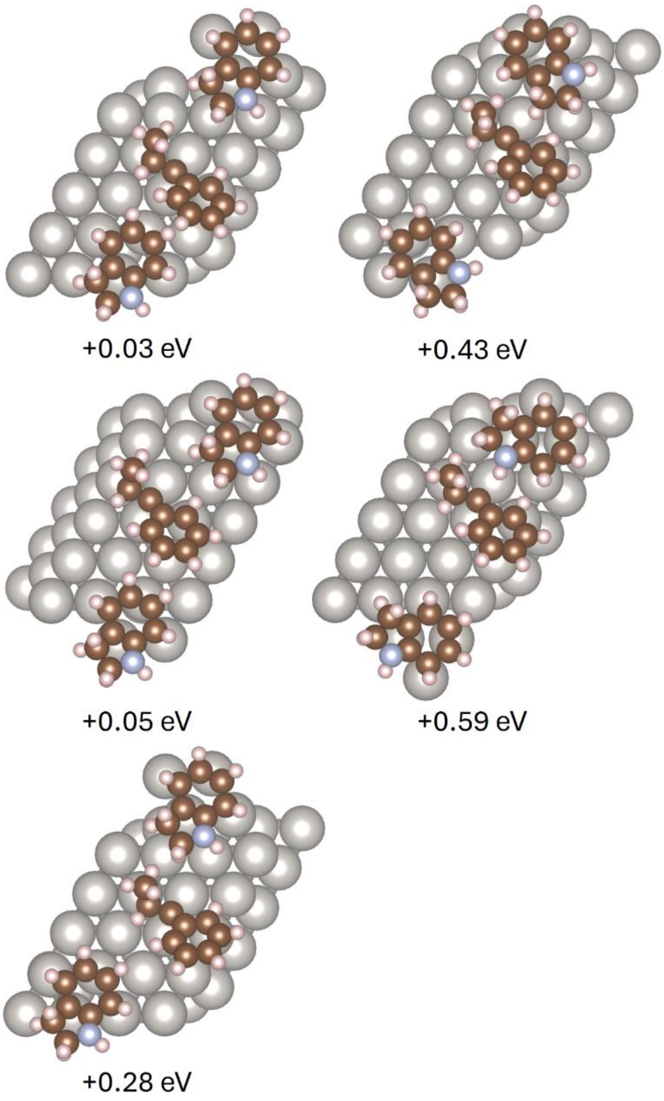

Figure S16. Alternative conformations for the starting state (**I**) of the *trans*-forming reaction complex with Ind (indoline) on Pt(111) surface. Energies are normalized on the lowest energy conformer.

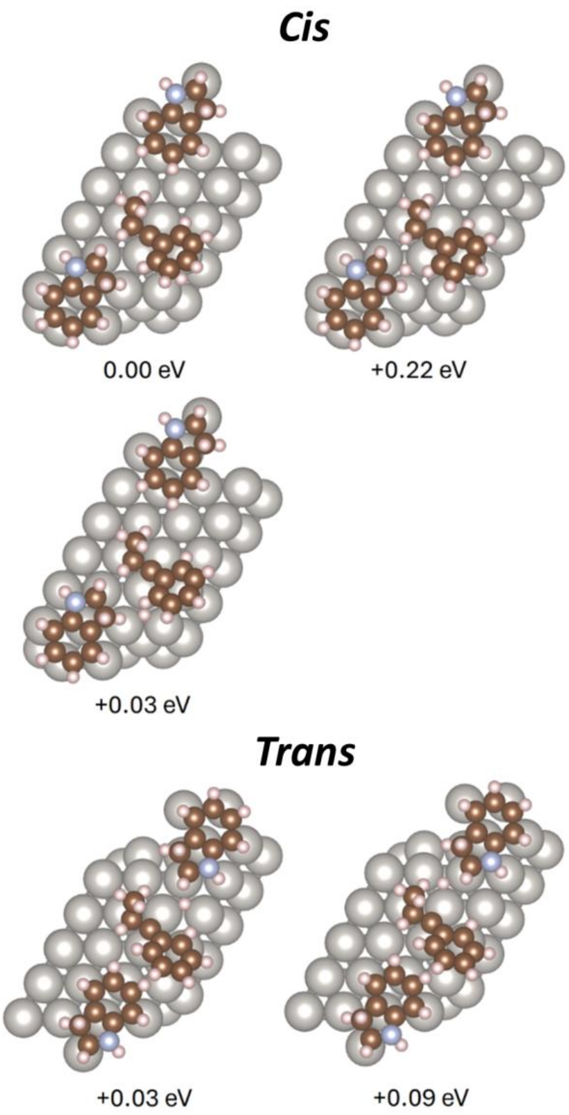

Figure S17. Alternative conformations for the intermediate state (**II**) of the reaction complex with Ind (Indoline) on Pt(111) surface. Energies are normalized on the lowest energy conformer.

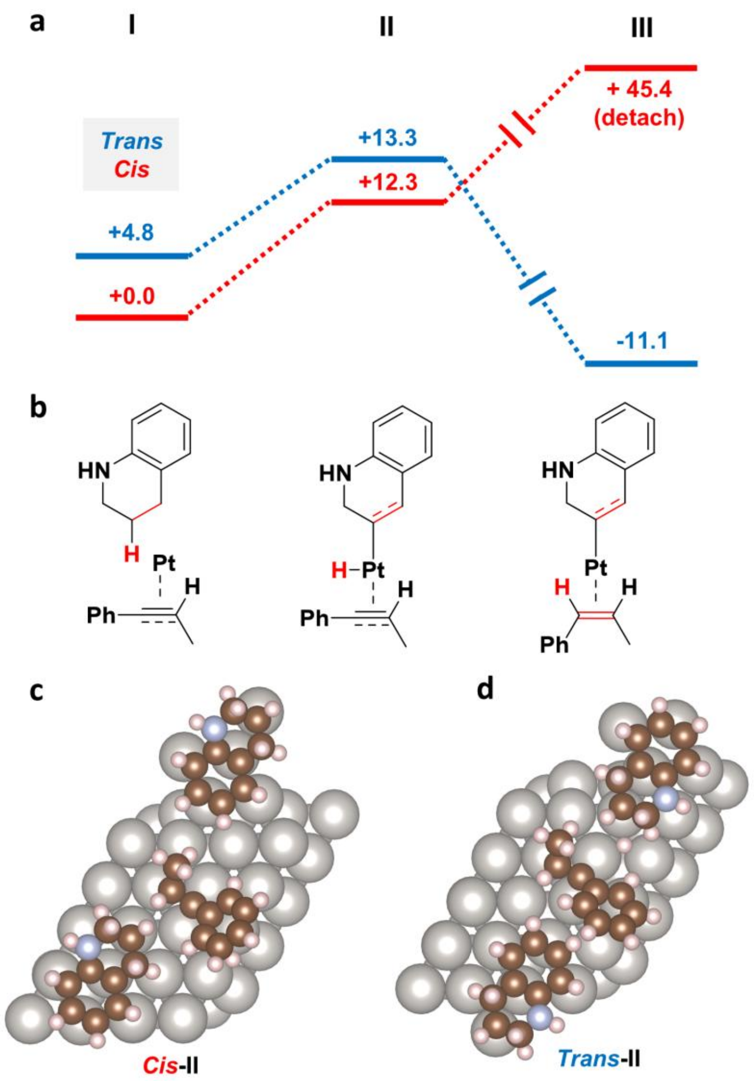

Figure S18. Lowest energy conformers of the first hydrogen transfer process forming the *cis*- and *trans*- alkenes. (**a**) Reactant state energy plot (all values are given in eV) with **I**. starting materials including Thq (tetrahydroquinoline) and alkyne **II**. Hydrogen transfer intermediates **III**. Product states. Notice that the relative distances across the energies are adjusted for clarity, but the relative magnitude was kept. (**b**) schematic representation of the reaction process. (**c**) Lowest energy *cis*-forming conformer. (**d**) Lowest energy *trans*-forming conformer.

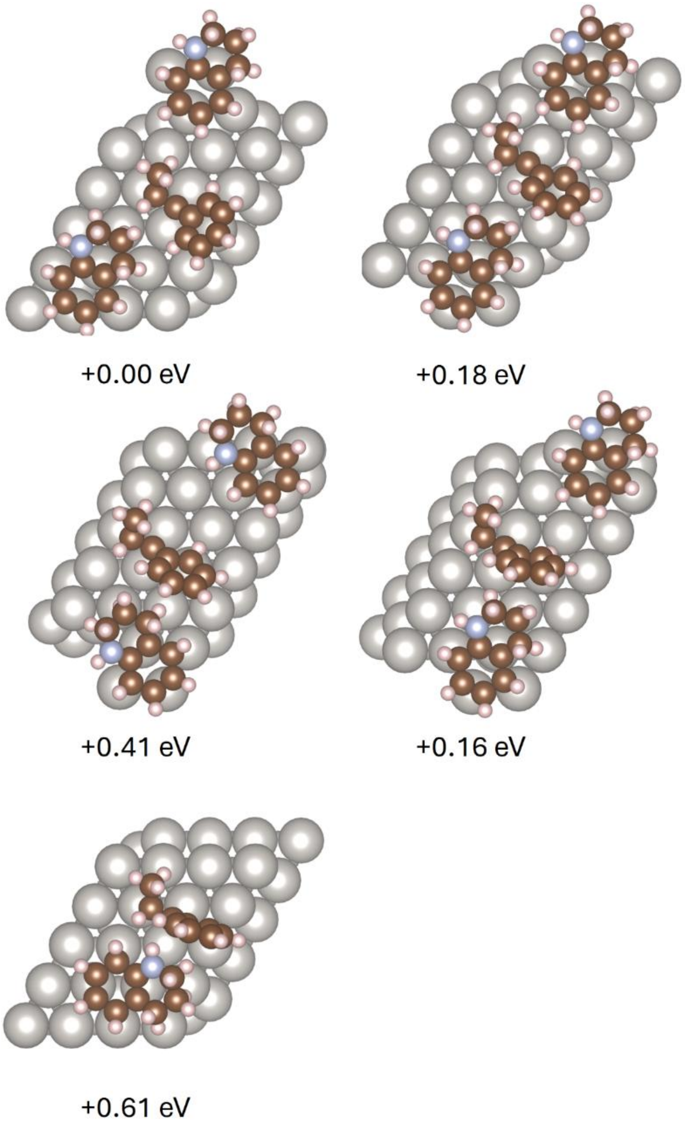

Figure S19. Alternative conformations for the starting state (**I**) of the *cis*-forming reaction complex with Thq (tetrahydroquinoline) on Pt(111) surface. Energies are normalized on the lowest energy conformer.

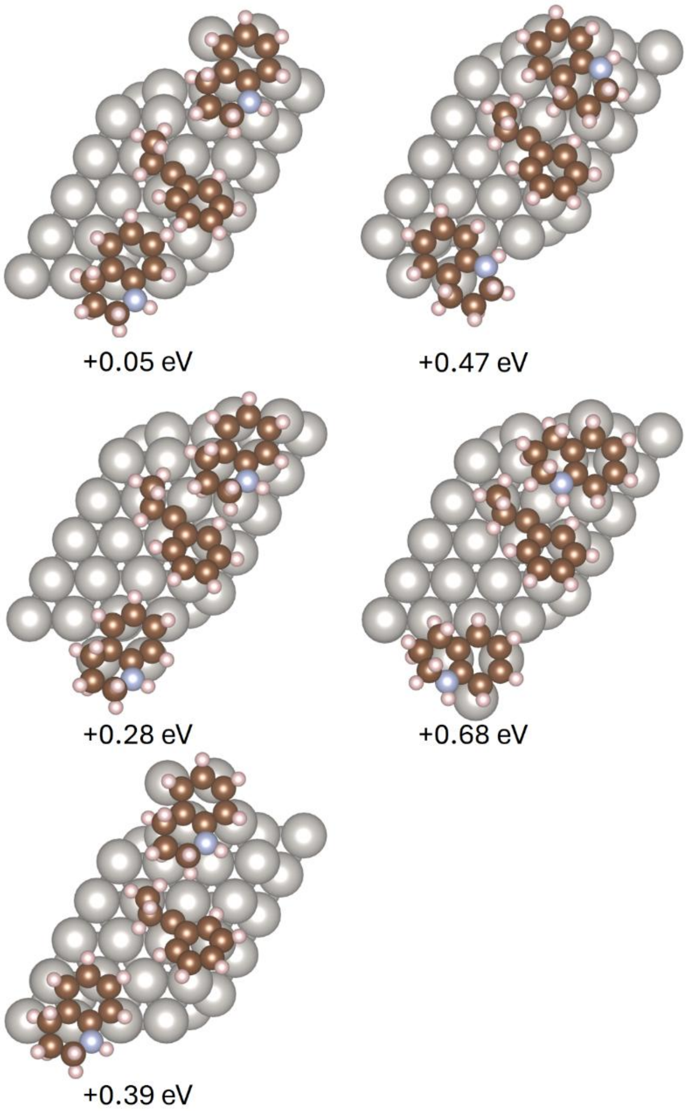

Figure S20. Alternative conformations for the starting state (**I**) of the *trans*-forming reaction complex with Thq (tetrahydroquinoline) on Pt(111) surface. Energies are normalized on the lowest energy conformer.

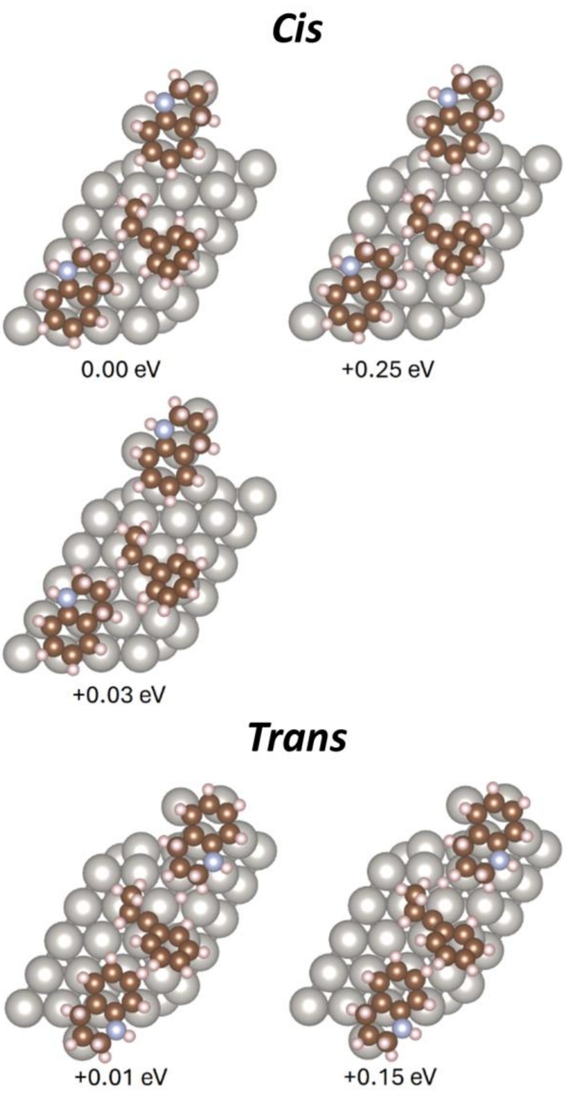

Figure S21. Alternative conformations for the intermediate state (**II**) of the reaction complex with Thq (tetrahydroquinoline) on Pt(111) surface. Energies are normalized on the lowest energy conformer.

Table S20. First dehydrogenation energies of the amine derivatives on Pt/C and Pd/C normalized on each corresponding N-site.

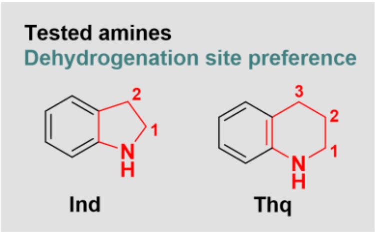

Adsorbate	Sites	Relative dehydrogenation energy (eV)	
		Pt(111)	Pd(111)
**Ind**	1	0.06	-0.08
	2	0.15	-0.14
**Thq**	1	-0.01	0.28
	2	-0.17	-0.12
	3	-0.04	-0.11

Table S21. Slab and gas phase energies for adsorbates.

Pure systems	Energy (eV)	Pure systems	Energy (eV)
**Substrate 1**	-131.50	**Ind**	-113.94
**Substrate 2**	-161.83	**HInd**	-106.50
**Substrate 3**	-109.28	**Thq**	-130.73
**(*cis*)-Alkene 1**	-140.40	**Quin**	-115.37
**(*cis*)-Alkene 2**	-170.49	**1,2-Di**	-122.64
**(*cis*)-Alkene 3**	-117.99	**2,4-Di**	-122.59
**(*trans*)-Alkene 1**	-140.58	**6x6x4 Pt**	-917.85
**(*trans*)-Alkene 2**	-170.66	**6x6x4 Pd**	-783.58
**(*trans*)-Alkene 3**	-118.08	-	-

Table S22. Binding complex energies for binding energies calculations.

Species +Slab	System energy (eV)	
	Pt(111)	Pd(111)
**Substrate 1**	-1053.14	-918.89
**Substrate 2**	-1084.01	-950.05
**Substrate 3**	-1030.73	-896.48
**(*cis*)-Alkene 1**	-1061.62	-927.45
**(*cis*)-Alkene 2**	-1092.12	-958.36
**(*cis*)-Alkene 3**	-1039.03	-904.76
**(*trans*)-Alkene 1**	-1062.04	-927.78
**(*trans*)-Alkene 2**	-1093.13	-959.15
**(*trans*)-Alkene 3**	-1039.56	-904.98
**Ind**	-1034.91	-900.49
**HInd**	-1027.42	-893.08
**Thq**	-1051.66	-917.33
**Quin**	-1036.50	-902.41
**1,2-Di**	-1043.67	-909.39
**2,4-Di**	-1044.28	-909.87

Table S23. Binding complex energies for the first hydrogen detachment.

Species +Slab	System energy (eV)	
	Pt(111)	Pd(111)
**Hydrogen atom**	-921.78	-787.68
**Ind pos 1**	-1030.91	-896.48
**Ind pos 2**	-1030.83	-896.53
**Thq pos 1**	-1047.73	-912.94
**Thq pos 2**	-1047.90	-913.35
**Thq pos 3**	-1047.67	-913.34

Table S24. Binding Complex Energies for reaction mechanism for Ind. All slabs are 4x5x4 Pt(111). (The order is consistent to **Figure S15** to **Figure S17.**)

Steps	*Cis*-forming	Energy (eV)	*Trans*-forming	Energy (eV)
**I**	**Conf 1**	-743.26	**Conf 1**	-743.22
	**Conf 2**	-743.17	**Conf 2**	-742.83
	**Conf 3**	-742.88	**Conf 3**	-743.20
	**Conf 4**	-743.12	**Conf 4**	-742.67
	**Conf 5**	-742.78	**Conf 5**	-742.98
**II**	**Conf 1**	-742.98	**Conf 1**	-742.94
	**Conf 2**	-742.76	**Conf 2**	-742.89
	**Conf 3**	-742.95		
**III**	**Conf 1**	-743.02	**Conf 1**	-743.44

Table S25. Binding Complex Energies for reaction mechanism for Thq. All slabs are 4x5x4 Pt(111). (The order is consistent to **Figure S19** to **Figure S21**.)

Steps	*Cis*-forming	Energy (eV)	*Trans*-forming	Energy (eV)
**I**	**Conf 1**	-759.98	**Conf 1**	-759.93
	**Conf 2**	-759.80	**Conf 2**	-759.49
	**Conf 3**	-759.57	**Conf 3**	-759.70
	**Conf 4**	-759.82	**Conf 4**	-759.30
	**Conf 5**	-759.37	**Conf 5**	-759.59
**II**	**Conf 1**	-759.85	**Conf 1**	-759.84
	**Conf 2**	-759.60	**Conf 2**	-759.70
	**Conf 3**	-759.82		
**III**	**Conf 1**	-759.51	**Conf 1**	-760.09

References

1 Roeder, G. J.; Kelly, R. H.; Yang, G.; Bauer, T. J.; Haller, G. L.; Batista, V. S.; Baráth, E. *ACS Catal.* **2021**, *11*, 5405.

2 Milakovic, L.; Hintermeier, P. H.; Y. Liu, Baráth, E.; Lercher, J. A. *Angew. Chem. Int. Ed.* **2021**, *60*, 24806.

3 Li, K. R. H. Kelly, R. H.; Franco, A.; Batista, V. S.; Baráth, E. *ACS Catal.* **2024**, *14*, 2883.

4 Kresse, G.; Furthmüller, J.; *Phys. Rev. B* **1996**, *54*, 11169.

5 Kresse, G.; Hafner, J. *Phys. Rev. B* **1993**, *47*, 558.

6 Kresse, G.; Hafner, J. *Phys. Rev. B* **1994**, *49*, 14251.

7 Kresse, G.; Joubert, D. *Phys. Rev. B* **1999**, *59*, 1758.

8 Kresse, G.; Furthmüller, J. *Comput. Mater. Sci.* **1996**, *6*, 15.

9 Grimme, S.; Antony, J.; Ehrlich, S.; Krieg, H. *J. Chem. Phys.* **2010**, *132*, 154104.

10 Grimme, S.; Ehrlich, S.; Goerigk, L. *J. Comput. Chem.* **2011**, *32*, 1456.

11 Monkhorst, H. J.; Pack, J. D. *Phys. Rev. B* **1976**, *13*, 5188.

12 Momma, K.; Izumi, F. *J. Appl. Crystallogr.* **2011**, *44*, 1272.

13 Frisch, M. J.; Trucks, G. W.; Schlegel, H. B.; Scuseria, G. E.; Robb, M. A.; Cheeseman, J. R.; Scalmani, G.; Barone, V.; Mennucci, B.; Petersson, G. A.; Nakatsuji, H.; Caricato, M.; Li, X.; Hratchian, H. P.; Izmaylov, A. F.; Bloino, J.; Zheng, G.; Sonnenberg, J. L.; Hada, M.; Ehara, M.; Toyota, K.; Fukuda, R.; Hasegawa, J.; Ishida, M.; Nakajima, T.; Honda, Y.; Kitao, O.; Nakai, H.; Vreven, T.; Montgomery Jr., J. A.; Peralta, J. E.; Ogliaro, F.; Bearpark, M.; Heyd, J. J.; Brothers, E.; Kudin, K. N.; Staroverov, V. N.; Keith, T.; Kobayashi, R.; Normand, J.; Raghavachari, K.; Rendell, A.; Burant, J. C.; Iyengar S. S.; Tomasi, J.; Cossi, M.; Rega, N.; Millam, J. M.; Klene, M.; Knox, J. E.; Cross, J. B.; Bakken, V.; Adamo, C.; Jaramillo, J.; Gomperts, R.; Stratmann, R. E.; Yazyev, O.; Austin, A. J.; Cammi, R.; Pomelli, C.; Ochterski, J. W.; Martin, R. L.; Morokuma, K.; Zakrzewski, V. G.; Voth, G. A.; Salvador, P.; Dannenberg, J. J.; Dapprich, S.; Daniels, A. D.; Farkas, O.; Foresman, J. B.; Ortiz, J. V.; Cioslowski, J.; Fox, D. J. Gaussian 09, Revision D.01, Gaussian, Inc., Wallingford CT. **2013**.

14 Dill, J. D.; Pople, J. A.; *J. Chem. Phys.* **1975**, *62*, 2921.

15 Hehre, W. J.; Ditchfield, R.; Pople, J. A. *J. Chem. Phys.* **1972**, *56*, 2257.

16 Lee, C.; Yang, W.; Parr, R. G. *Phys. Rev. B* **1988**, *37*, 785–789.

17 Mennucci, B. *Wiley Interdiscip. Rev. Comput. Mol. Sci.* **2012**, *2*, 386.

18 Mulliken, R. S. *Phys. Rev.* **1932**, *41*, 749.

19 Keith, T. A.; Dennington, R.; Millam, J. M. GaussView, Version 6.1, Semichem Inc., Shawnee Mission, KS, **2016**.
